# The double burden of malnutrition in under-five children at national and individual levels: observed and expected prevalence in ninety-three low- and middle-income countries

**DOI:** 10.1017/S1368980020001226

**Published:** 2020-07-07

**Authors:** Beatriz Raffi Lerm, Inácio Crochemore-Silva, Janaína Calu Costa, Cesar Gomes Victora

**Affiliations:** International Center for Equity in Health, Post-Graduate Program in Epidemiology, Federal University of Pelotas, Pelotas, RS 96020-220, Brazil

**Keywords:** Double burden, Malnutrition, Health inequalities, Stunting, Overweight

## Abstract

**Objective:**

To assess whether the observed prevalence of the double burden of malnutrition (DBM) would be higher than expected on the basis of chance, through analyses at national, wealth quintile and individual child levels.

**Design:**

We selected nationally representative surveys from low- and middle-income countries (LMIC) carried out since 2005 with anthropometric measures on children under 5 years of age. Household wealth was assessed through asset indices. The expected prevalence of DBM was estimated by multiplying the prevalence of stunting (low height/length for age) and overweight (high weight for height/length). The WHO recommended cut-offs (20% for stunting and 10% for overweight) that were used to define DBM at national level. DBM at individual level was defined as co-occurrence of stunting and overweight in the same child.

**Setting:**

Nationally representative surveys from ninety-three LMIC.

**Participants:**

A total of 825 633 children were studied.

**Results:**

DBM at national level was observed in five countries, whereas it would be expected to occur in eleven countries. Six countries did not present evidence of DBM at national level but did so in at least one wealth quintile. At individual level, thirty countries (32·3%) showed higher prevalence of DBM than would be expected, but most differences were small except for Syria, Azerbaijan, Albania and Egypt.

**Conclusions:**

The observed number of countries or socio-economic subgroups within countries with the DBM using recommended thresholds was below what would be expected by chance. However, individual-level analyses showed that one-third of countries presented higher prevalence of DBM than would be expected.

The coexistence of contrasting extremes of the malnutrition spectrum, such as stunting and overweight, is known as the double burden of malnutrition (DBM). This phenomenon^(1,2)^ has been described at the individual level, when the same person presents both short stature and overweight^(3)^, at household level, when stunting and overweight affect different members (often when children present undernutrition and their mothers are overweight)^(4,5)^ and at the population level, when prevalence of both stunting and overweight is high within the same population group^(6)^. Regarding the latter definition, the 2019 Joint Child Malnutrition Estimates Report^(7)^ highlighted the lack of estimates on the proportion of children who are affected by both stunting and overweight.

International organisations state that the DBM poses a novel public health challenge for low- and middle-income countries (LMIC)^(2)^, where high levels of undernutrition persist and overweight is on the rise^(1,3)^. While stunting is strongly related to poverty, resulting from poor diets, inadequate care and repeated infections^(8)^, the association between overweight and family wealth is less clear in LMIC settings^(6,9–13)^.

The DBM at population level has been described by several authors, with variable definitions^(1,5,11–13)^. For children under 5 years of age, a national prevalence of stunting above 20% or a prevalence of overweight above 10% has been suggested as indicating public health concerns^(14,15)^. In the current analyses, we describe how many LMIC with recent surveys present both problems, using these criteria.

However, even if a given country presents high prevalence of both stunting and overweight, these problems may be affecting different subgroups of the population, for example, stunting being common among children from poor families and overweight affecting those from wealthy families. Our second set of analyses investigate the prevalence of each condition by household’s wealth quintiles in LMIC.

Our third set of analyses address the request by the 2019 Joint Child Malnutrition Estimates Report^(7)^ for estimates of the DBM at the individual child level. Rather than rely solely on the prevalence of stunting and overweight^(16–18)^, our approach also recognises that the overlap between the two conditions may occur due to chance. For instance, Dieffenbach & Stein^(17)^, studying the co-occurrence of stunted children and overweight mothers within each household in 112 surveys from fifty-four countries, found little evidence of such a ‘double burden’ combination once chance was accounted for. We applied the same approach to compare our estimate at individual level with what would be predicted by chance alone.

The current analyses contribute to the literature by taking into account that the DBM, both at population and at individual levels, may be present due to chance. This requires estimating the expected prevalence at each level and comparing it with observed prevalence. This will allow a critical assessment of present estimates of the magnitude of double burden at individual and population levels.

## Methods

### Data sources and measurements

The current analyses are based on Demographic and Health Surveys^(19)^ and Multiple Indicators Clusters Surveys^(20)^. These are nationally representative surveys which collect data on reproductive, maternal, neonatal and child health and nutrition in LMIC. Both surveys are similarly designed, using multistage cluster sampling and standardised questionnaires and data collection procedures, allowing therefore comparability of results^(21)^.

Publicly available data from Demographic and Health Surveys and Multiple Indicators Clusters Surveys have been processed and reanalysed by the International Center for Equity in Health (ICEH) at the Federal University of Pelotas (Brazil) to provide disaggregated estimates that enable the study of social and economic inequalities. The ICEH database currently comprises 224 Demographic and Health Surveys and 116 Multiple Indicators Clusters Surveys from 114 countries. The current analyses included the most recent Demographic and Health Surveys or Multiple Indicators Clusters Surveys from each country carried out from 2005 to 2017, in which anthropometric data were available for children under 5 years of age.

For the anthropometric assessment, measuring boards were used to obtain recumbent length for children under 2 years of age and standing height for the older children. Weight was measured using portable digital scales. Children under 2 years of age were weighted while on an adult’s lap, and afterwards the weight of the adult was subtracted^(22,23)^. Stunting prevalence was defined as the proportion of children presenting height/length for age below –2 sd relative to the median of the WHO Child Growth Standards^(24)^. Overweight prevalence was defined as the proportion of children with weight for height/length above +2 sd from the median of the reference curve for their age^(24)^. Ages of the children were estimated by subtracting the birth date (reported by the mother or caregiver) from the interview date; some older surveys only had data on month of birth, and the 15th of each month was used as the day of birth. Children who presented both stunting and overweight were considered as suffering from the DBM at individual level.

Wealth indices were calculated at household level for each survey, based on a score of assets, home construction materials and facilities (e.g., water and electricity) obtained through principal components analyses^(25)^. The first component resulting from these analyses was divided into quintiles, with the first representing the poorest 20% of all households in the sample, and the fifth representing the wealthiest 20%^(26)^. Considering that fertility rates and household sizes are larger among the poor, the proportion of children tends to be slightly above 20% in the poorest quintiles and below 20% in the richest quintiles. Cuba and Djibouti were excluded from wealth-specific analyses due to lack of information on variables needed to estimate the asset index.

### Data analyses

Descriptive analyses include estimates of the DBM at individual level and of the prevalence of stunting and overweight at national level and for wealth quintiles. Point estimates and 95% CI are presented. Results are summarised by world regions and country’s income groups, using the median value and interquartile range (IQR) for the three outcomes. The UNICEF classification was used to characterise seven regions: West and Central Africa, Eastern and Southern Africa, Middle East and North Africa, Europe and Central Asia, South Asia, East Asia and the Pacific and Latin America and the Caribbean. Income groups were based on the World Bank’s classification, which takes into account the country’s gross national income *per capita* in 2017–2018 as follows: low income (gross national income ≤$1005), lower-middle income (gross national income $1006–$3955) and upper-middle income (gross national income $3956–$12 235)^(27,28)^.

Correlations between the prevalence of stunting and overweight at national level and in each wealth quintile, region and income group were assessed using Spearman’s rank-order coefficients. This method was chosen, given the asymmetric distribution of the stunting and overweight estimates.

All data were analysed using Stata (Stata Statistical Software: Release 15; StataCorp LLC), taking into account the complex sampling structure of the surveys and the sampling weights. All the data used are publicly available, and ethical clearance for data collection was under the responsibility of the national institutions that administered the surveys.

### Characterisation of the double burden

We used two definitions of DBM, one for population-level and another for individual-level analyses. At population level, the number of countries presenting the DBM was estimated based on thresholds of high prevalence suggested by international organisations, namely, prevalence above 20 and 10% for stunting and overweight, respectively^(14,15)^. The expected number of countries with DBM was estimated using by multiplying the proportion of countries with high stunting prevalence by the proportion with high overweight prevalence. The same approach was also used to assess the DBM within wealth quintiles.

For individual-level analyses, the observed estimates of the DBM were compared with the expected probabilities of children presenting both stunting and overweight due to chance (assuming that the two conditions were independent from one another) using the formula:

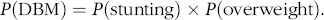




The variance for the expected DBM prevalence was calculated as follows:

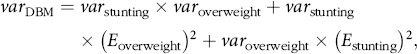

where *E* = estimate and *var* = se ^2^(*n*). The variance estimate was used to calculate 95% CI.

The observed DBM prevalence at individual level was reported as higher than expected when the lower limit of its 95% CI was above the upper limit of the 95% CI for the expected prevalence. By subtracting the observed prevalence by the expected prevalence, we assessed the magnitude of the excess of DBM at individual level in each country, expressed in percentage points.

## Results

Ninety-three LMIC had eligible surveys carried out between 2005 and 2017 (median year was 2013), which were included in the analyses. Twenty-six low-income, forty-three lower-middle- and twenty-four upper-middle-income countries were assessed. The median number of children per survey was 5481 (IQR 2895–9165), and the total number of children was 825 633.

### Analyses at national level

Stunting prevalence varied from 2·5% (95% CI 1·2, 5·2) in St. Lucia to 55·9% (95% CI 54·2, 57·7) in Burundi, whilst overweight prevalence ranged from 0·9% (95% CI 0·8, 1·2) in Senegal to 22·3% (95% CI 19·5, 26·0) in Montenegro. Results by country are available in the online supplementary material, Supplemental Table 1.

Globally, the median prevalence was 25·5% (IQR 12·9–35·3) for stunting, 5·2% (IQR 2·8–8·2) for overweight and 1·4% (IQR 0·9–2·6) for both conditions present in the same child. Differences among country’s income groups and world regions are presented in Table 1. Stunting prevalence was highest in low-income countries, whereas overweight was most frequent in upper-middle-income countries. Double burden prevalence did not vary among country’s income groups but was highest in the Middle East and North Africa (3·4%) and the lowest in Latin America and the Caribbean (0·6%).

**Table 1:** Correlation and prevalence of stunting and overweight and double burden at individual level, globally, by country’s income group and world region in low- and middle-income countries with a survey since 2005

Group	*n*	Stunting (%)			Overweight (%)			Correlation between stunting and overweight†		Double burden at individual level (%)		
		Median	P25	P75	Median	P25	P75	*ρ*	*P*	Median	P25	P75
All countries	93	25·5	12·9	35·3	5·2	2·8	8·2	−0·51	<0·001	1·4	0·9	2·6
Low-income countries	26	34·5	30·1	39·9	2·8	1·9	4·5	0·13	0·52	1·4	0·9	2·2
Lower-middle-income countries	43	26·2	14·6	36·1	5·2	2·6	8·5	−0·42	0·01	1·5	0·7	3·1
Upper-middle-income countries	24	11·6	8·4	17·3	7·8	6·0	12·7	−0·16	0·45	1·4	0·8	3·6
		<0·001*			<0·001*					0·928		
East Asia and the Pacific	8	27·5	16·7	38·3	4·6	2·0	6·8	−0·50	0·21	1·1	0·8	2·0
Eastern and Southern Africa	16	36·2	30·6	39·2	4·7	3·5	7·4	−0·22	0·41	2·1	1·3	3·1
Europe and Central Asia	16	9·4	6·2	16·9	12·3	6·4	15·6	0·19	0·47	2·7	1·4	5·0
Latin America and Caribbean	15	13·2	8·8	21·8	6·3	4·8	7·3	−0·28	0·32	0·6	0·5	1·1
Middle East and North Africa	10	21·7	10·1	32·5	11·9	4·4	14·3	−0·19	0·60	3·4	1·3	7·2
South Asia	6	36·0	33·5	38·0	2·8	1·4	6·0	−0·37	0·47	1·4	0·7	2·3
West and Central Africa	22	31·0	21·8	37·9	2·4	1·8	4·0	−0·01	0·98	1·1	0·8	1·8
		<0·001*			<0·001*					<0·001*		

* *P*-level (Kruskal–Wallis test) comparing prevalence of the outcomes among country’s income groups and world regions.

† Spearman’s correlation coefficient between stunting and overweight prevalence within country’s income groups and world regions, with countries as the units of analyses.

Spearman’s correlation coefficients were calculated within each group of countries. Using all countries as the units of analysis, there was a moderate inverse correlation between stunting and overweight prevalence (*r* –0·51; *P* < 0·001; Table 1). Despite the inverse correlation between stunting and overweight at global level, the two outcomes were not correlated within low-income countries (*r* 0·13; *P* = 0·519), nor within upper-middle-income countries (*r* –0·16; *P* = 0·450). The inverse correlation was only observed within lower-middle-income countries (*r* –0·42; *P* = 0·005; see Table 1 and online supplementary material, Supplemental Fig. 1). Correlations within each region were non-significant and difficult to interpret due to the small number of countries in each group (Table 1).

**Fig. 1:**
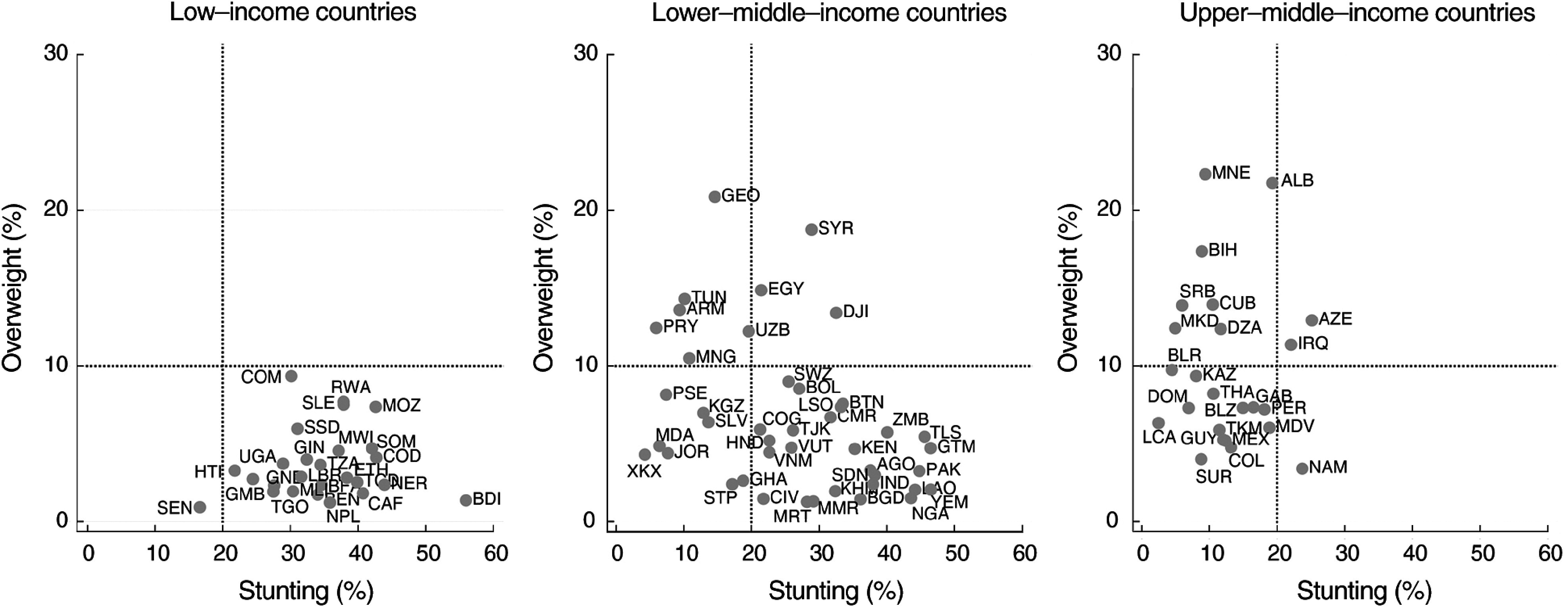
Countries according to their stunting and overweight prevalence

Table 2 shows the number of countries where the DBM was expected and the number where it was present. Five of the ninety-three countries had point estimates for both stunting and overweight prevalence above the international thresholds (Table 2 and Fig. 1), whereas eleven countries would be expected to present DBM by chance alone (Table 2). In all five countries (Azerbaijan, Djibouti, Egypt, Iraq and Syria), the lower limits of the 95% CI for the national estimates were above the international thresholds for both stunting and overweight (see online supplementary material, Supplemental Table 1 for the CI). In summary, there was evidence of regional variability in DBM prevalence, but it did not vary by country income, and the number of countries affected was lower than what would be expected by chance.

**Table 2:** Expected and observed numbers of countries and wealth quintiles presenting the double burden of malnutrition

	National		Poorest		Second		Middle		Fourth		Wealthiest	
	*n*	%	*n*	%	*n*	%	*n*	%	*n*	%	*n*	%
Expected double burden	11	11·9	10	10·8	11	11·8	9	9·4	11	11·6	7	7·5
Observed double burden	5	5·4	6	6·6	5	5·5	3	3·3	5	5·5	5	5·5

At national level, ninety-three countries were assessed. In each wealth quintiles, ninety-one countries were assessed.

### Analyses by wealth quintile

To address whether DBM may be affecting children from different socio-economic strata within the same country, analyses by wealth quintile were performed. Table 3 is complementary to Table 2, by showing the five countries with evidence of DBM, as well as another six countries where there was no such evidence at national level, but where DBM was present in at least one wealth quintile. In Syria, the DBM was present at national level and in all quintiles, while in Egypt, it was present in four quintiles (the exception being the middle quintile). In Azerbaijan and Uzbekistan, the DBM was observed in the three poorest quintiles. Some countries did not present evidence of DBM at national level but did so in at least one quintile: Albania, Bhutan, Comoros, Georgia, Rwanda and Uzbekistan. Summing up, there was no evidence that countries with DBM presented a combination of stunting among children from poor households and overweight among those from wealthy families.

**Table 3:** Countries with point estimates for stunting ≥20% and overweight ≥10% at national level and/or wealth quintile levels

National	Poorest	Second	Middle	Fourth	Wealthiest
	Albania			Albania	
Azerbaijan	Azerbaijan	Azerbaijan	Azerbaijan		
					Bhutan
				Comoros	
Djibouti					
Egypt	Egypt	Egypt		Egypt	Egypt
	Georgia				
Iraq		Iraq		Iraq	Iraq
					Rwanda
Syria	Syria	Syria	Syria	Syria	Syria
	Uzbekistan	Uzbekistan	Uzbekistan		

### Analyses at individual level

The last set of analyses refer to the presence of DBM at individual rather than country or quintile level. The last columns in Table 1 show that the median national values of DBM prevalence did not vary by country’s income groups.

**Fig. 2:**
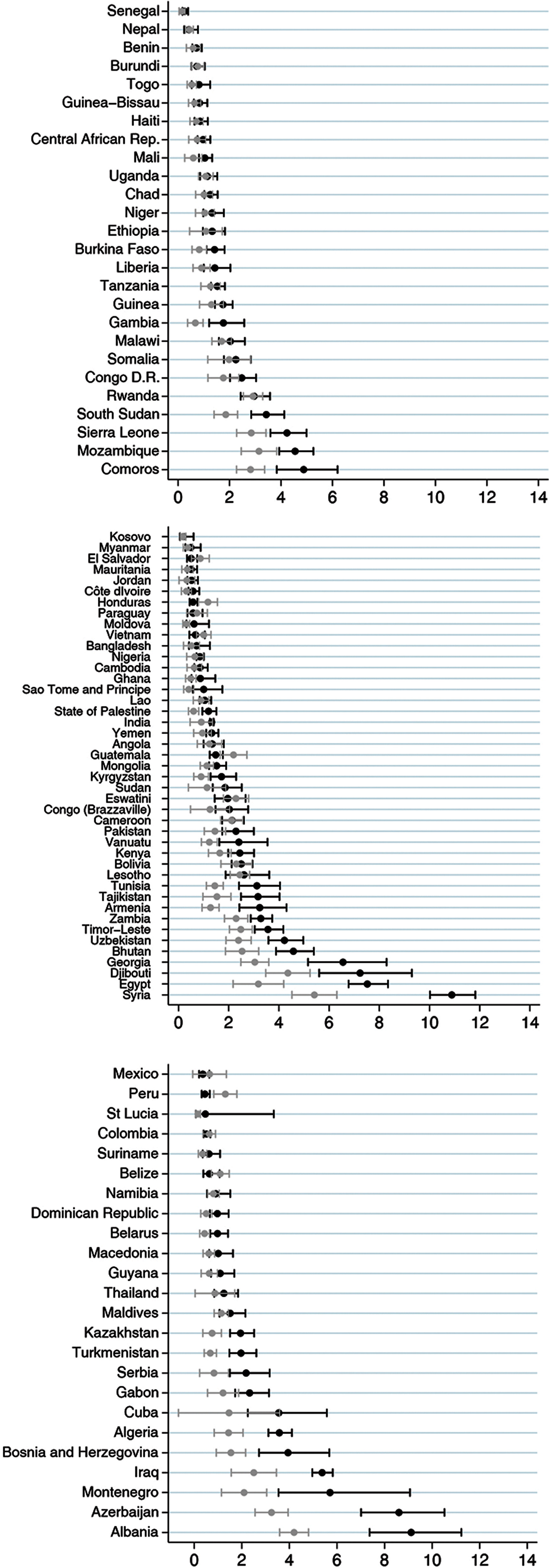
Countries observed and expected prevalence of double burden of malnutrition at individual level and its respective CI, by World Bank income groups

Fig. 2 shows the individual-level analyses by country. The proportion of children with DBM ranged from 0·2% (95% CI 0·1, 0·6) in Kosovo to 10·9% (95% CI 10·0, 11·8) in Syria. A total of thirty countries (32·3%) presented the lower limit value of the 95% CI for the observed prevalence above the upper limit of the expected prevalence (Fig. 2), and only in Peru and Honduras, the observed prevalence was significantly lower than expected. Eighteen of these countries had a difference between the observed and expected values of <2 percentage points. The highest differences were observed in countries with high DBM prevalence – Syria, Azerbaijan, Albania and Egypt – where the observed prevalence was >4 percentage points compared with the expected value.

## Discussion

We reported on the prevalence of stunting, overweight and of both conditions among children under 5 years of age through analyses at individual and population levels. The analyses by country’s income groups confirm the inverse association between national wealth and stunting prevalence and its direct association with overweight prevalence. In contrast, DBM prevalence was 1·5% or lower in most income or regional groups. Still in the country-level analyses, stunting prevalence was inversely and moderately correlated with overweight prevalence, at national level for all ninety-three countries, but this association was driven by lower-middle-income countries, where the number of stunted children dropped rapidly and that of overweight children increased markedly from 2000 to 2017^(15)^. As shown in the online supplementary material, Supplemental Fig. 1, regardless of the frequency of stunting, overweight tends to be rare in low-income countries and high in upper-middle-income countries. Only lower-middle-income countries seem to have sufficient heterogeneity in both indicators to result in a significant, inverse correlation. It should be noted that our correlation analyses are simply descriptive, and there is no attempt to assess causality. In addition, the cross-sectional nature of the current analyses, based on the most recent available surveys, do not reflect trends over time. Nevertheless, the presence of DBM likely reflects the ongoing nutrition transition in a given setting^(2)^.

Still at national level, the number of countries presenting the DBM (defined as stunting prevalence ≥ 20% and overweight prevalence ≥ 10%) was lower than what would be expected by chance. Only five countries – including four of the ten countries from the Middle East and North Africa region in our analyses – presented evidence of the DBM at national level. It should be noted that we do not have data for North African countries such as Algeria, Libya, Morocco and Tunisia, where this problem may also be present. We found a single study that attempted to identify LMIC where the DBM was present among children under 5 years of age at national level, rather than at individual level. Tzioumis *et al*.^(13)^ analysed thirty-six countries and defined that the DBM was a ratio of stunting prevalence to overweight prevalence close to 1·0. Only four countries (Armenia, Egypt, Dominican Republic and Jordan) had ratios below 1·5 and were identified as presenting DBM using this criterion. Of the above countries, Egypt was classified by our analyses as presenting the DBM at national level, and Dominican Republic was not; Armenia and Jordan were not studied. The lack of national studies on DBM prevalence at population level is likely due to the fact that although the population prevalence threshold of 20% for defining stunting as a public health problem has been available for decades^(29)^, only recently the 10% cut-off for overweight prevalence was proposed by international agencies^(14,15)^.

Next, we examined whether children from a particular wealth quintile were more likely to be affected by DBM. Two studies – from Ghana^(30)^ and Mexico^(31)^ – reported higher prevalence of the double burden among children from the poorest quintiles. Nevertheless, our analyses did not support these findings (Table 3), as no clear patterns of DBM by wealth were found. We did not find any similar analyses in the published literature.

A critique of most of analyses of DBM is lack of recognition that, due to chance alone, stunting and overweight may coexist at population, household or individual level^(17)^. Examples of such analyses include the recent Lancet series on DBM (data in the web annex)^(5)^, the above-cited article by Tzioumis *et al*.^(13)^ and the work of Bates *et al*.^(32)^ who estimated DBM at individual level (which they refer to as ‘stuntedoverweight’ children) in seventy-nine LMIC, with prevalence ranging from 0·3% in Senegal to 11·7% in Guinea-Bissau.

In our analyses, national prevalence of the DBM at individual level ranged from 0·2% to 10·9%, which is consistent with the literature^(13,32–34)^ and also consistent with the overall estimate 2·0% (ranged from 0·0% in Niue to 10·1% in Egypt) from the Global School-Based Student Health and Health Behavior in School-Aged Children surveys conducted in fifty-seven LMIC^(35)^. In thirty of the ninety-three countries in our analyses, more children presented DBM than would be expected by chance, although in most countries, the excess was rather small. The largest excesses were observed in countries from Sub-Saharan Africa, the Middle East and in Eastern Europe and Central Asia.

A few articles considered the expected prevalence when assessing the co-occurrence of stunting and overweight in children. The observed prevalence was lower than the expected in children aged 5–11 years from Ecuador^(11)^ and among children aged 5–12 years from Colombia^(36)^. In contrast, a study of Indian children aged 6–59 months reported that in twenty-five of the thirty-six states studied, the observed DBM prevalence was significantly higher than would be expected^(37)^.

Our approach enhances the above analyses by also accounting for the variability not only around the observed prevalence but also around the expected DBM prevalence. Additional strengths of our analyses include the large number of countries studied and the use of nationally representative surveys which are highly comparable^(21)^.

The limitations of our analyses include the fact that surveys were spread out over several years (from 2005 up to 2017) and the fact that a large proportion of the surveys come from low-income and lower-middle-income countries, with more restricted representation of upper-middle-income or high-income countries. To assess whether the inclusion of older surveys would affect the results, we restricted the analyses to eighty-one surveys carried out in 2010 or later. The proportion of countries with national-level evidence of the DBM falls from 5·4% (all surveys) to 2·5% (more recent surveys). Likewise, evidence of DBM at individual level falls from 32·3 to 27·2%, respectively. These reductions were mostly due to declines in the prevalence of stunting over time. Thus, there is no evidence of higher DBM prevalence in newer surveys. We did not exclude countries affected by humanitarian crises, such as Syria and Yemen, because we aimed at describing the situation in as many countries as possible. In addition, there is no agreement on a precise definition for ‘conflict’ nor on the length of post-conflict period during which populations may still experience its consequences^(38)^. Results on these countries should be interpreted while bearing in mind their special conditions, as in the short-term humanitarian emergencies are likely to affect overweight, and in the long term both stunting and overweight prevalence.

It is important to note that quintiles are relative measures of socio-economic position and that, for example, the poorest quintile in a given country could be similar in terms of wealth to say the third quintile in another country. Despite this limitation, the descriptive analyses by quintiles were important to address the possibility that, within each country, DBM would be more common in a particular socio-economic group. In addition, several authors postulate that relative poverty is as important in predicting deprivation^(39)^ and health status^(40)^ as is absolute poverty.

Another limitation is that the expected prevalence of DBM was calculated assuming the independence of stunting and overweight^(42)^. However, both events are possibly biologically related^(43)^ and may share common determinants and mechanisms including the microbiome, hormonal regulation and lean and fat mass growth patterns^(44)^. In addition, stunting and overweight may be related mathematically, since height – used to evaluate stunting – is also in the denominator of weight for height/length, which is utilised to characterise overweight. By assuming that the two conditions are independent, we opted for a conservative approach in calculating the expected prevalence levels. Indeed, our finding that in many countries the observed prevalence is somewhat higher than the expected level may be an indication that the two conditions are indeed biologically related. Finally, our results on DBM prevalence are limited to young children, whereas overweight^(41)^ and likely DBM prevalence increase with age.

Recently, the Lancet Series on the DBM addressed the dynamics of DBM, its aetiological pathways and consequences for health, its economics effects and programmatic and policy implications^(5,44–46)^. A broad definition of DBM was adopted, including presence of stunting among children, overweight among children and adults, as well as micronutrient deficiencies. None of the analyses in this series took into account the expected prevalence of DBM. The series provides a thorough review of the causes of the DBM, such as early life nutrition, diet diversity, food environments and socio-economic factors. It advocates for so-called double-duty actions against DBM^(45)^ at multiple levels, including health services, social safety nets, educational settings and agriculture, food systems and food environments.

Summing up, we provide a comprehensive overview of the occurrence of the DBM in children at population and individual levels. We find that few countries and few wealth groups within countries present evidence of a DBM epidemic. The main conclusion from the individual level analyses is that in one-third of the countries, the proportions of children presenting both stunting and overweight are slightly higher than would be expected by change. Continued monitoring of DBM prevalence at national and individual levels is essential for assessing the progress of the epidemic and for providing guidance to national and international policies.

## Supplementary material

For supplementary material accompanying this paper visit https://doi.org/10.1017/S1368980020001226. click here to view supplementary material

## References

[1] Tzioumis E & Adair LS (2014) Childhood dual burden of under- and overnutrition in low- and middle-income countries: a critical review. Food Nutr Bull 35, 230–243. 25076771 10.1177/156482651403500210 PMC4313560

[2] World Health Organization (WHO) (2017) Double-Duty Actions for Nutrition: Policy Brief. Geneva: WHO.

[3] Pomeroy E, Stock JT, Stanojevic S et al. (2014) Stunting, adiposity, and the individual-level “dual burden” among urban lowland and rural highland Peruvian children. Am J Hum Biol 26, 481–490. 24706334 10.1002/ajhb.22551 PMC4312888

[4] Kosaka S & Umezaki M (2017) A systematic review of the prevalence and predictors of the double burden of malnutrition within households. Br J Nutr 117, 1118–1127. 28514987 10.1017/S0007114517000812

[5] Popkin BM, Corvalan C & Grummer-Strawn LM (2020) Dynamics of the double burden of malnutrition and the changing nutrition reality. Lancet 395, 65–74. 31852602 10.1016/S0140-6736(19)32497-3 PMC7179702

[6] Black RE, Victora CG, Walker SP et al. (2013) Maternal and child undernutrition and overweight in low-income and middle-income countries. Lancet 382, 427–451. 23746772 10.1016/S0140-6736(13)60937-X

[7] United Nations International Children’s Emergency Fund (UNICEF), World Health Organization (WHO) & International Bank for Reconstruction and Development – World Bank (2019) Levels and Trends in Child Malnutrition: Joint Child Malnutrition Estimates: Key Findings of the 2019 Edition. New York: UNICEF.

[8] De Onis M & Branca F (2016) Childhood stunting: a global perspective. Matern Child Nutr 12(S1), 12–26. 27187907 10.1111/mcn.12231 PMC5084763

[9] Food and Agriculture Organization of the United Nations (2006) The double burden of malnutrition: case studies from six developing countries. FAO Food Nutr Pap 84, 1–334. 19172986

[10] Development Initiatives Poverty Research (2018) Global Nutrition Report: Shining a Light to Spur Action on Nutrition. Bristol: Development Initiatives.

[11] Freire WB, Silva-Jaramillo KM, Ramirez-Luzuriaga MJ et al. (2014) The double burden of undernutrition and excess body weight in Ecuador. Am J Clin Nutr 100, 1636S–1643S. 25411306 10.3945/ajcn.114.083766

[12] Le Nguyen BK, Le Thi H, Nguyen Do VA et al. (2013) Double burden of undernutrition and overnutrition in Vietnam in 2011: results of the SEANUTS study in 0.5–11-year-old children. Br J Nutr 110, Suppl. 3, S45–S56. 24016766 10.1017/S0007114513002080

[13] Tzioumis E, Kay MC, Bentley ME et al. (2016) Prevalence and trends in the childhood dual burden of malnutrition in low- and middle-income countries, 1990–2012. Public Health Nutr 19, 1375–1388. 26905921 10.1017/S1368980016000276 PMC5470367

[14] De Onis M, Borghi E, Arimond M et al. (2018) Prevalence thresholds for wasting, overweight and stunting in children under 5 years. Public Health Nutr 22, 175–179. 30296964 10.1017/S1368980018002434 PMC6390397

[15] United Nations International Children’s Emergency Fund (UNICEF), World Health Organization (WHO) & World Bank Group (2018) Levels and Trends in Child Malnutrition: Key Findings of the 2018 Edition of the Joint Child Malnutrition Estimates. New York: UNICEF.

[16] Corsi DJ, Finlay JE & Subramanian SV (2011) Global burden of double malnutrition: has anyone seen it? PLoS One 6, e25120. 21980384 10.1371/journal.pone.0025120 PMC3182195

[17] Dieffenbach S & Stein AD (2012) Stunted child/overweight mother pairs represent a statistical artifact, not a distinct entity. J Nutr 142, 771–773. 22378330 10.3945/jn.111.153387

[18] Jehn M & Brewis A (2009) Paradoxical malnutrition in mother-child pairs: untangling the phenomenon of over- and under-nutrition in underdeveloped economies. Econ Hum Biol 7, 28–35. 19246260 10.1016/j.ehb.2009.01.007

[19] U.S. Agency for International Development (USAID) (2018) The Demographic and Health Surveys (DHS). Calverton, MD: USAID.

[20] United Nations International Children’s Emergency Fund (UNICEF) (2018) Multiple Indicator Cluster Surveys (MICS). New York: UNICEF.

[21] Hancioglu A & Arnold F (2013) Measuring coverage in MNCH: tracking progress in health for women and children using DHS and MICS household surveys. PLoS Med 10, e1001391. 23667333 10.1371/journal.pmed.1001391 PMC3646216

[22] United Nations International Children’s Emergency Fund (UNICEF) (2017) MICS Manual for Anthropometry. New York: UNICEF.

[23] U.S. Agency for International Development (USAID) (2012) Biomarker Field Manual, Demographic and Health Surveys Methodology. Calverton, MD: USAID.

[24] World Health Organization (WHO) (2006) WHO Child Growth Standards: Length/Height for Age, Weight-for-Age, Weight-for-Length, Weight-for-Height and Body Mass Index-for-Age, Methods and Development. Geneva: WHO.

[25] Barros AJD & Victora CG (2013) Measuring coverage in MNCH: determining and interpreting inequalities in coverage of maternal, newborn, and child health interventions. PLoS Med 10, e1001390. 23667332 10.1371/journal.pmed.1001390 PMC3646214

[26] Howe LD, Galobardes B, Matijasevich A et al. (2012) Measuring socio-economic position for epidemiological studies in low-and middle-income countries: a methods of measurement in epidemiology paper. Int J Epidemiol 41, 871–886. 22438428 10.1093/ije/dys037 PMC3396323

[27] United Nations International Children’s Emergency Fund (UNICEF) (2017) Country, Regional and Divisional Annual Reports. New York: UNICEF.

[28] World Bank Group (2018) World Bank Country and Lending Groups. New York: World Bank.

[29] Ferro-Luzzi A, Garza C, Haas J et al. (1995) Physical Status: The Use and Interpretation of Anthropometry. Geneva: World Health Organization.

[30] Atsu BK, Guure C & Laar AK (2017) Determinants of overweight with concurrent stunting among Ghanaian children. BMC Pediatr 17, 177. 28750614 10.1186/s12887-017-0928-3 PMC5531099

[31] Fernald LC & Neufeld LM (2007) Overweight with concurrent stunting in very young children from rural Mexico: prevalence and associated factors. Eur J Clin Nutr 61, 623–632. 17136036 10.1038/sj.ejcn.1602558

[32] Bates K, Gjonca A & Leone T (2017) Double burden or double counting of child malnutrition? The methodological and theoretical implications of stunting overweight in low and middle income countries. J Epidemiol Community Health 71, 779–785. 28566281 10.1136/jech-2017-209008 PMC5537509

[33] Piernas C, Wang D, Du S et al. (2015) The double burden of under- and overnutrition and nutrient adequacy among Chinese preschool and school-aged children in 2009–2011. Eur J Clin Nutr 69, 1323. 26130296 10.1038/ejcn.2015.106 PMC4668216

[34] Rachmi CN, Agho KE, Li M et al. (2016) Stunting coexisting with overweight in 2·0–4·9-year-old Indonesian children: prevalence, trends and associated risk factors from repeated cross-sectional surveys. Public Health Nutr 19, 2698–2707. 27120968 10.1017/S1368980016000926 PMC5039402

[35] Caleyachetty R, Thomas G, Kengne AP et al. (2018) The double burden of malnutrition among adolescents: analysis of data from the global school-based student health and health behavior in school-aged children surveys in 57 low-and middle-income countries. Am J Clin Nutr 108, 414–424. 29947727 10.1093/ajcn/nqy105

[36] Sarmiento OL, Parra DC, Gonzalez SA et al. (2014) The dual burden of malnutrition in Colombia. Am J Clin Nutr 100, 1628S–1635S. 25411305 10.3945/ajcn.114.083816

[37] Varghese JS & Stein AD (2019) Malnutrition among women and children in India: limited evidence of clustering of underweight, anemia, overweight, and stunting within individuals and households at both state and district levels. Am J Clin Nutr 109, 1207–1215. 30882139 10.1093/ajcn/nqy374

[38] Bhutta ZA, Yousafzai AK & Zipursky A (2010) Pediatrics, war, and children. Curr Probl Pediatr Adolesc Health Care 40, 20–35. 20152786 10.1016/j.cppeds.2009.12.004

[39] Sen A (1992) Inequality Reexamined. Oxford: Oxford University Press.

[40] Marmot M (2017) The health gap: the challenge of an unequal world: the argument. Int J Epidemiol 46, 1312–1318. 28938756 10.1093/ije/dyx163 PMC5837404

[41] Cole TJ, Bellizzi MC, Flegal KM et al. (2000) Establishing a standard definition for child overweight and obesity worldwide: international survey. BMJ 320, 1240. 10797032 10.1136/bmj.320.7244.1240 PMC27365

[42] Bove I, Miranda T, Campoy C et al. (2012) Stunting, overweight and child development impairment go hand in hand as key problems of early infancy: Uruguayan case. Early Hum Dev 88, 747–751. 22560815 10.1016/j.earlhumdev.2012.04.002

[43] Barker DJP (1998) Mothers, Babies and Health in Later Life. London: Elsevier Health Sciences.

[44] Wells JC & Victora CG (2005) Indices of whole-body and central adiposity for evaluating the metabolic load of obesity. Int J Obes 29, 483–489. 10.1038/sj.ijo.0802899 15672103

[45] Hawkes C, Ruel MT, Salm L et al. (2020) Double-duty actions: seizing programme and policy opportunities to address malnutrition in all its forms. Lancet 395, 142–155. 31852603 10.1016/S0140-6736(19)32506-1

[46] Nugent R, Levin C, Hale J et al. (2020) Economic effects of the double burden of malnutrition. Lancet 395, 156–164. 31852601 10.1016/S0140-6736(19)32473-0

