# Supplementary material for: The double burden of malnutrition in under-five children at national and individual levels: observed and expected prevalence in ninety-three low- and middle-income countries
Source: Public Health Nutr. 2020 Jul 7;24(10):2944–51. doi: 10.1017/S1368980020001226 (PMC7613087; doi:10.1017/S1368980020001226)

**Supplementary Table 1.** Prevalence of stunting, overweight and double burden at individual level, in children under five years from LMIC, according to each country.

| **Country** | **Year** | **World region (UNICEF)** | **Stunting** | | | **Overweight** | | | **Double burden at individual level** | | | **Expected prevalence*** | | | **Significant individual double burden †** | **Sample size** |
| --- | --- | --- | --- | --- | --- | --- | --- | --- | --- | --- | --- | --- | --- | --- | --- | --- |
|  |  |  | **%** | **95%CI** | | **%** | **95%CI** | | **%** | **95%CI** | | **%** | **95%CI** | |  |  |
| **Low-income countries** | | | | | | | | | | | | | | | | |
| Benin | 2014 | West & Central Africa | 34.0 | 32.7 | 35.4 | 1.7 | 1.5 | 2.0 | 0.7 | 0.5 | 0.9 | 0.6 | 0.3 | 0.9 | no | 11,991 |
| Burkina Faso | 2010 | West & Central Africa | 34.6 | 33.2 | 36.1 | 2.4 | 1.9 | 2.9 | 1.4 | 1.1 | 1.8 | 0.8 | 0.5 | 1.1 | yes | 6,991 |
| Burundi | 2016 | Eastern & Southern Africa | 55.9 | 54.2 | 57.7 | 1.4 | 1.1 | 1.7 | 0.7 | 0.5 | 1.0 | 0.8 | 0.5 | 1.0 | no | 6,455 |
| Central African Rep. | 2010 | West & Central Africa | 40.7 | 39.1 | 42.4 | 1.8 | 1.5 | 2.2 | 1.0 | 0.8 | 1.3 | 0.7 | 0.4 | 1.1 | no | 10,214 |
| Chad | 2014 | West & Central Africa | 39.9 | 38.4 | 41.3 | 2.5 | 2.2 | 2.9 | 1.2 | 1.0 | 1.5 | 1.0 | 0.7 | 1.3 | no | 10,852 |
| Comoros | 2012 | Eastern & Southern Africa | 30.1 | 27.9 | 32.4 | 9.3 | 7.9 | 11.0 | 4.9 | 3.8 | 6.2 | 2.8 | 2.3 | 3.4 | yes | 2,761 |
| Congo D.R. | 2013 | West & Central Africa | 42.7 | 40.9 | 44.5 | 4.1 | 3.5 | 4.8 | 2.5 | 2.0 | 3.0 | 1.8 | 1.2 | 2.4 | no | 9,028 |
| Ethiopia | 2016 | Eastern & Southern Africa | 38.4 | 36.5 | 40.3 | 2.8 | 2.3 | 3.5 | 1.3 | 1.0 | 1.8 | 1.1 | 0.4 | 1.7 | no | 10,376 |
| Gambia | 2013 | West & Central Africa | 24.5 | 22.4 | 26.7 | 2.7 | 2.1 | 3.6 | 1.8 | 1.2 | 2.6 | 0.7 | 0.4 | 1.0 | yes | 3,369 |
| Guinea | 2016 | West & Central Africa | 32.4 | 31.0 | 33.9 | 4.0 | 3.3 | 4.9 | 1.7 | 1.4 | 2.1 | 1.3 | 0.8 | 1.8 | no | 7,080 |
| Guinea-Bissau | 2014 | West & Central Africa | 27.6 | 26.2 | 29.0 | 2.3 | 2.0 | 2.8 | 0.8 | 0.6 | 1.1 | 0.6 | 0.4 | 0.9 | no | 7,446 |
| Haiti | 2016 | Latin America & Caribbean | 21.8 | 20.3 | 23.3 | 3.3 | 2.8 | 3.8 | 0.9 | 0.6 | 1.2 | 0.7 | 0.5 | 1.0 | no | 6,568 |
| Liberia | 2013 | West & Central Africa | 31.6 | 29.3 | 34.0 | 2.9 | 2.2 | 3.7 | 1.4 | 1.0 | 2.0 | 0.9 | 0.6 | 1.2 | no | 3,520 |
| Malawi | 2015 | Eastern & Southern Africa | 37.1 | 35.6 | 38.7 | 4.5 | 3.9 | 5.3 | 2.0 | 1.6 | 2.6 | 1.7 | 1.3 | 2.1 | no | 5,707 |
| Mali | 2015 | West & Central Africa | 30.4 | 29.2 | 31.7 | 1.9 | 1.6 | 2.3 | 1.0 | 0.8 | 1.3 | 0.6 | 0.3 | 0.9 | no | 14,872 |
| Mozambique | 2011 | Eastern & Southern Africa | 42.6 | 41.0 | 44.2 | 7.4 | 6.7 | 8.1 | 4.5 | 3.9 | 5.3 | 3.1 | 2.4 | 3.8 | yes | 10,313 |
| Nepal | 2016 | South Asia | 35.8 | 33.5 | 38.3 | 1.2 | 0.9 | 1.7 | 0.4 | 0.2 | 0.8 | 0.4 | 0.3 | 0.6 | no | 2,421 |
| Niger | 2012 | West & Central Africa | 43.9 | 41.9 | 45.9 | 2.4 | 1.9 | 3.0 | 1.3 | 1.0 | 1.8 | 1.0 | 0.7 | 1.4 | no | 5,481 |
| Rwanda | 2014 | Eastern & Southern Africa | 37.9 | 36.1 | 39.6 | 7.7 | 6.9 | 8.6 | 2.9 | 2.4 | 3.6 | 2.9 | 2.5 | 3.3 | no | 3,813 |
| Senegal | 2017 | West & Central Africa | 16.6 | 15.7 | 17.6 | 0.9 | 0.7 | 1.2 | 0.3 | 0.2 | 0.4 | 0.2 | 0.0 | 0.3 | no | 10,933 |
| Sierra Leone | 2013 | West & Central Africa | 37.9 | 35.9 | 39.8 | 7.5 | 6.6 | 8.6 | 4.2 | 3.6 | 5.0 | 2.8 | 2.3 | 3.4 | yes | 5,094 |
| Somalia | 2006 | Eastern & Southern Africa | 42.1 | 39.4 | 44.8 | 4.7 | 3.8 | 5.8 | 2.2 | 1.8 | 2.8 | 2.0 | 1.2 | 2.8 | no | 5,700 |
| South Sudan | 2010 | Eastern & Southern Africa | 31.1 | 29.4 | 32.8 | 6.0 | 5.2 | 6.8 | 3.4 | 2.8 | 4.1 | 1.9 | 1.4 | 2.3 | yes | 5,736 |
| Tanzania | 2015 | Eastern & Southern Africa | 34.4 | 33.0 | 35.9 | 3.6 | 3.2 | 4.1 | 1.5 | 1.3 | 1.8 | 1.3 | 0.9 | 1.6 | no | 9,846 |
| Togo | 2013 | West & Central Africa | 27.5 | 25.7 | 29.4 | 1.9 | 1.5 | 2.5 | 0.8 | 0.5 | 1.2 | 0.5 | 0.4 | 0.7 | no | 3,279 |
| Uganda | 2016 | Eastern & Southern Africa | 28.9 | 27.3 | 30.5 | 3.7 | 3.2 | 4.4 | 1.1 | 0.8 | 1.5 | 1.1 | 0.8 | 1.4 | no | 5,117 |
| **Lower-middle-income countries** | | | | | | | | | | | | | | | | |
| Angola | 2015 | Eastern & Southern Africa | 37.6 | 35.7 | 39.5 | 3.3 | 2.7 | 4.0 | 1.3 | 1.0 | 1.8 | 1.2 | 0.8 | 1.7 | no | 7,388 |
| Armenia | 2015 | Europe & Central Asia | 9.4 | 7.8 | 11.3 | 13.6 | 11.4 | 16.1 | 3.2 | 2.4 | 4.3 | 1.3 | 0.9 | 1.6 | yes | 1,573 |
| Bangladesh | 2014 | South Asia | 36.1 | 34.4 | 37.9 | 1.4 | 1.1 | 1.9 | 0.7 | 0.4 | 1.3 | 0.5 | 0.2 | 0.8 | no | 7,317 |
| Bhutan | 2010 | South Asia | 33.5 | 31.6 | 35.5 | 7.6 | 6.6 | 8.7 | 4.6 | 3.9 | 5.4 | 2.5 | 1.9 | 3.2 | yes | 5,805 |
| Bolivia | 2008 | Latin America & Caribbean | 27.1 | 25.5 | 28.7 | 8.5 | 7.7 | 9.4 | 2.5 | 2.1 | 2.9 | 2.3 | 1.7 | 2.9 | no | 8,416 |
| Cambodia | 2014 | East Asia & the Pacific | 32.4 | 30.6 | 34.2 | 2.0 | 1.5 | 2.6 | 0.9 | 0.6 | 1.2 | 0.6 | 0.3 | 0.9 | no | 4,892 |
| Cameroon | 2014 | West & Central Africa | 31.7 | 30.2 | 33.3 | 6.7 | 6.0 | 7.5 | 2.1 | 1.7 | 2.6 | 2.1 | 1.7 | 2.6 | no | 6,726 |
| Congo (Brazzaville) | 2014 | West & Central Africa | 21.3 | 19.9 | 22.7 | 5.9 | 4.8 | 7.2 | 2.0 | 1.5 | 2.8 | 1.3 | 0.5 | 2.0 | no | 8,696 |
| Côte dIvoire | 2016 | West & Central Africa | 21.8 | 20.6 | 23.0 | 1.5 | 1.2 | 1.9 | 0.6 | 0.4 | 0.8 | 0.3 | 0.1 | 0.5 | no | 8,937 |
| Djibouti | 2006 | Middle East & North Africa | 32.5 | 29.2 | 36.0 | 13.4 | 11.2 | 15.9 | 7.2 | 5.6 | 9.3 | 4.4 | 3.5 | 5.2 | yes | 2,070 |
| Egypt | 2014 | Middle East & North Africa | 21.4 | 20.1 | 22.9 | 14.9 | 13.9 | 15.9 | 7.5 | 6.8 | 8.3 | 3.2 | 2.2 | 4.2 | yes | 13,598 |
| El Salvador | 2014 | Latin America & Caribbean | 13.6 | 12.5 | 14.9 | 6.4 | 5.6 | 7.2 | 0.5 | 0.3 | 0.7 | 0.9 | 0.5 | 1.2 | no | 7,220 |
| Eswatini | 2014 | Eastern & Southern Africa | 25.5 | 23.5 | 27.6 | 9.0 | 7.5 | 10.7 | 2.0 | 1.4 | 2.7 | 2.3 | 1.8 | 2.8 | no | 1,859 |
| Georgia | 2005 | Europe & Central Asia | 14.6 | 12.5 | 17.0 | 20.8 | 18.6 | 23.3 | 6.6 | 5.2 | 8.3 | 3.0 | 2.5 | 3.6 | yes | 2,895 |
| Ghana | 2014 | West & Central Africa | 18.8 | 17.0 | 20.6 | 2.6 | 2.0 | 3.5 | 0.9 | 0.5 | 1.5 | 0.5 | 0.3 | 0.7 | no | 12,567 |
| Guatemala | 2014 | Latin America & Caribbean | 46.5 | 44.8 | 48.2 | 4.7 | 4.3 | 5.2 | 1.5 | 1.2 | 1.8 | 2.2 | 1.7 | 2.7 | no | 10,165 |
| Honduras | 2011 | Latin America & Caribbean | 22.7 | 21.5 | 23.9 | 5.2 | 4.7 | 5.8 | 0.6 | 0.4 | 0.8 | 1.2 | 0.8 | 1.6 | no | 219,796 |
| India | 2015 | South Asia | 38.0 | 37.7 | 38.3 | 2.4 | 2.3 | 2.5 | 1.3 | 1.3 | 1.4 | 0.9 | 0.5 | 1.4 | no | 5,851 |
| Jordan | 2012 | Middle East & North Africa | 7.7 | 6.6 | 8.9 | 4.4 | 3.6 | 5.3 | 0.5 | 0.4 | 0.8 | 0.3 | 0.0 | 0.6 | no | 5,470 |
| Kenya | 2008 | Eastern & Southern Africa | 35.3 | 33.2 | 37.4 | 4.7 | 4.0 | 5.4 | 2.4 | 2.0 | 3.0 | 1.6 | 1.2 | 2.1 | no | 1,513 |
| Kosovo | 2013 | Europe & Central Asia | 4.3 | 3.2 | 5.7 | 4.3 | 3.3 | 5.5 | 0.2 | 0.1 | 0.6 | 0.2 | 0.1 | 0.3 | no | 4,415 |
| Kyrgyzstan | 2014 | Europe & Central Asia | 12.9 | 11.7 | 14.2 | 7.0 | 6.1 | 8.0 | 1.7 | 1.3 | 2.3 | 0.9 | 0.6 | 1.2 | yes | 10,685 |
| Lao | 2011 | East Asia & the Pacific | 44.2 | 42.6 | 45.7 | 2.0 | 1.7 | 2.4 | 1.0 | 0.8 | 1.3 | 0.9 | 0.6 | 1.2 | no | 1,869 |
| Lesotho | 2014 | Eastern & Southern Africa | 33.2 | 30.7 | 35.9 | 7.4 | 6.1 | 8.8 | 2.6 | 1.9 | 3.6 | 2.4 | 2.1 | 2.8 | no | 10,110 |
| Mauritania | 2015 | West & Central Africa | 28.2 | 26.9 | 29.6 | 1.3 | 1.0 | 1.6 | 0.5 | 0.3 | 0.7 | 0.4 | 0.1 | 0.6 | no | 1,704 |
| Moldova | 2012 | Europe & Central Asia | 6.4 | 5.3 | 7.8 | 4.9 | 3.8 | 6.2 | 0.6 | 0.3 | 1.2 | 0.3 | 0.2 | 0.4 | no | 5,725 |
| Mongolia | 2013 | East Asia & the Pacific | 10.8 | 9.9 | 11.8 | 10.5 | 9.6 | 11.4 | 1.5 | 1.2 | 1.9 | 1.1 | 0.9 | 1.4 | no | 4,088 |
| Myanmar | 2015 | East Asia & the Pacific | 29.1 | 27.3 | 31.1 | 1.3 | 0.9 | 1.8 | 0.5 | 0.3 | 0.9 | 0.4 | 0.2 | 0.6 | no | 27,320 |
| Nigeria | 2016 | West & Central Africa | 43.6 | 42.4 | 44.7 | 1.5 | 1.3 | 1.7 | 0.9 | 0.7 | 1.0 | 0.7 | 0.3 | 1.0 | no | 3,465 |
| Pakistan | 2012 | South Asia | 44.8 | 42.1 | 47.5 | 3.2 | 2.5 | 4.1 | 2.3 | 1.7 | 3.0 | 1.5 | 1.0 | 1.9 | no | 4,402 |
| Paraguay | 2016 | Latin America & Caribbean | 5.9 | 4.9 | 7.2 | 12.4 | 11.0 | 14.1 | 0.6 | 0.4 | 1.0 | 0.7 | 0.3 | 1.1 | no | 1,931 |
| Sao Tome and Principe | 2014 | West & Central Africa | 17.2 | 15.2 | 19.4 | 2.4 | 1.6 | 3.5 | 1.0 | 0.6 | 1.8 | 0.4 | 0.2 | 0.6 | no | 6,950 |
| State of Palestine | 2014 | Middle East & North Africa | 7.4 | 6.7 | 8.2 | 8.2 | 7.4 | 8.9 | 1.2 | 1.0 | 1.5 | 0.6 | 0.4 | 0.8 | yes | 11,333 |
| Sudan | 2014 | Middle East & North Africa | 38.2 | 36.5 | 40.0 | 3.0 | 2.4 | 3.7 | 1.9 | 1.4 | 2.5 | 1.1 | 0.4 | 1.9 | no | 2,618 |
| Syria | 2006 | Middle East & North Africa | 28.9 | 27.6 | 30.2 | 18.7 | 17.6 | 19.9 | 10.9 | 10.0 | 11.8 | 5.4 | 4.5 | 6.3 | yes | 10,307 |
| Tajikistan | 2012 | Europe & Central Asia | 26.2 | 24.4 | 28.0 | 5.9 | 4.8 | 7.1 | 3.2 | 2.5 | 4.0 | 1.5 | 1.0 | 2.1 | yes | 5,080 |
| Timor-Leste | 2016 | East Asia & the Pacific | 45.6 | 44.0 | 47.2 | 5.5 | 4.8 | 6.2 | 3.6 | 3.0 | 4.2 | 2.5 | 2.0 | 2.9 | yes | 6,714 |
| Tunisia | 2011 | Middle East & North Africa | 10.1 | 8.7 | 11.7 | 14.3 | 12.8 | 16.0 | 3.1 | 2.4 | 4.0 | 1.4 | 1.1 | 1.8 | yes | 2,589 |
| Uzbekistan | 2006 | Europe & Central Asia | 19.6 | 18.1 | 21.1 | 12.2 | 11.0 | 13.5 | 4.2 | 3.6 | 5.0 | 2.4 | 1.9 | 2.9 | yes | 4,821 |
| Vanuatu | 2007 | East Asia & the Pacific | 25.9 | 22.7 | 29.4 | 4.7 | 3.6 | 6.2 | 2.4 | 1.6 | 3.5 | 1.2 | 0.9 | 1.5 | yes | 1,313 |
| Vietnam | 2010 | East Asia & the Pacific | 22.7 | 20.9 | 24.5 | 4.4 | 3.7 | 5.3 | 0.7 | 0.4 | 1.0 | 1.0 | 0.7 | 1.3 | no | 3,572 |
| Yemen | 2013 | Middle East & North Africa | 46.5 | 45.1 | 47.9 | 2.1 | 1.8 | 2.4 | 1.3 | 1.1 | 1.6 | 1.0 | 0.6 | 1.3 | no | 13,821 |
| Zambia | 2013 | Eastern & Southern Africa | 40.1 | 38.9 | 41.3 | 5.7 | 5.2 | 6.3 | 3.3 | 2.9 | 3.7 | 2.3 | 1.8 | 2.8 | yes | 12,456 |
| **Upper-middle-income countries** | | | | | | | | | | | | | | | | |
| Albania | 2008 | Europe & Central Asia | 19.3 | 16.8 | 22.1 | 21.7 | 18.8 | 25.0 | 9.1 | 7.4 | 11.2 | 4.2 | 3.6 | 4.8 | yes | 1,287 |
| Algeria | 2012 | Middle East & North Africa | 11.7 | 10.8 | 12.6 | 12.4 | 11.5 | 13.3 | 3.6 | 3.1 | 4.1 | 1.4 | 0.8 | 2.1 | yes | 13,736 |
| Azerbaijan | 2006 | Europe & Central Asia | 25.1 | 22.2 | 28.3 | 12.9 | 11.0 | 15.1 | 8.6 | 7.0 | 10.5 | 3.2 | 2.6 | 3.9 | yes | 1,573 |
| Belarus | 2005 | Europe & Central Asia | 4.5 | 3.5 | 5.6 | 9.7 | 8.6 | 11.0 | 1.0 | 0.7 | 1.4 | 0.4 | 0.2 | 0.6 | yes | 3,029 |
| Belize | 2015 | Latin America & Caribbean | 14.9 | 13.0 | 17.1 | 7.3 | 6.0 | 8.9 | 0.6 | 0.4 | 1.1 | 1.1 | 0.7 | 1.5 | no | 2,412 |
| Bosnia and Herzegovina | 2011 | Europe & Central Asia | 8.9 | 6.9 | 11.3 | 17.4 | 15.0 | 20.0 | 3.9 | 2.7 | 5.7 | 1.5 | 0.9 | 2.2 | yes | 2,137 |
| Colombia | 2010 | Latin America & Caribbean | 13.2 | 12.5 | 13.9 | 4.8 | 4.4 | 5.2 | 0.5 | 0.4 | 0.7 | 0.6 | 0.4 | 0.9 | no | 15,696 |
| Cuba | 2014 | Latin America & Caribbean | 10.5 | 8.0 | 13.6 | 13.9 | 11.4 | 17.0 | 3.6 | 2.2 | 5.6 | 1.5 | -0.7 | 3.6 | no | 5,375 |
| Dominican Republic | 2013 | Latin America & Caribbean | 6.9 | 6.0 | 8.1 | 7.3 | 6.2 | 8.6 | 1.0 | 0.7 | 1.4 | 0.5 | 0.3 | 0.7 | no | 3,618 |
| Gabon | 2012 | West & Central Africa | 16.5 | 14.3 | 18.9 | 7.3 | 6.0 | 9.0 | 2.3 | 1.7 | 3.1 | 1.2 | 0.6 | 1.9 | no | 3,855 |
| Guyana | 2014 | Latin America & Caribbean | 12.0 | 10.3 | 14.0 | 5.3 | 4.2 | 6.5 | 1.1 | 0.7 | 1.7 | 0.6 | 0.3 | 1.0 | no | 3,057 |
| Iraq | 2011 | Middle East & North Africa | 22.1 | 21.3 | 22.8 | 11.4 | 10.7 | 12.0 | 5.4 | 5.0 | 5.8 | 2.5 | 1.6 | 3.5 | yes | 35,040 |
| Kazakhstan | 2015 | Europe & Central Asia | 8.0 | 7.0 | 9.2 | 9.3 | 8.1 | 10.8 | 1.9 | 1.5 | 2.5 | 0.8 | 0.4 | 1.1 | yes | 5,277 |
| Macedonia | 2011 | Europe & Central Asia | 4.9 | 3.7 | 6.6 | 12.4 | 10.2 | 15.1 | 1.0 | 0.6 | 1.6 | 0.6 | 0.4 | 0.9 | no | 1,318 |
| Maldives | 2009 | South Asia | 18.9 | 16.9 | 20.9 | 6.0 | 5.0 | 7.2 | 1.5 | 1.1 | 2.1 | 1.1 | 0.8 | 1.4 | no | 2,513 |
| Mexico | 2015 | Latin America & Caribbean | 12.4 | 10.6 | 14.3 | 5.2 | 4.3 | 6.2 | 0.4 | 0.2 | 0.6 | 0.6 | -0.1 | 1.4 | no | 7,802 |
| Montenegro | 2013 | Europe & Central Asia | 9.4 | 6.6 | 13.2 | 22.3 | 18.4 | 26.7 | 5.7 | 3.5 | 9.1 | 2.1 | 1.1 | 3.0 | yes | 1,361 |
| Namibia | 2013 | Eastern & Southern Africa | 23.7 | 21.7 | 25.9 | 3.4 | 2.6 | 4.4 | 0.9 | 0.5 | 1.5 | 0.8 | 0.6 | 1.1 | no | 2,281 |
| Peru | 2012 | Latin America & Caribbean | 18.1 | 17.0 | 19.3 | 7.2 | 6.4 | 8.1 | 0.4 | 0.3 | 0.7 | 1.3 | 0.8 | 1.8 | no | 9,164 |
| Serbia | 2014 | Europe & Central Asia | 6.0 | 4.5 | 7.9 | 13.9 | 11.2 | 17.1 | 2.2 | 1.5 | 3.2 | 0.8 | 0.2 | 1.4 | yes | 2,337 |
| St Lucia | 2012 | Latin America & Caribbean | 2.5 | 1.2 | 5.2 | 6.3 | 3.4 | 11.4 | 0.5 | 0.1 | 3.3 | 0.2 | 0.1 | 0.3 | no | 279 |
| Suriname | 2010 | Latin America & Caribbean | 8.8 | 7.6 | 10.2 | 4.0 | 3.2 | 5.1 | 0.6 | 0.4 | 1.1 | 0.4 | 0.2 | 0.5 | no | 2,744 |
| Thailand | 2015 | East Asia & the Pacific | 10.6 | 9.3 | 12.0 | 8.2 | 7.2 | 9.4 | 1.2 | 0.8 | 1.8 | 0.9 | 0.0 | 1.7 | no | 11,022 |
| Turkmenistan | 2015 | Europe & Central Asia | 11.5 | 10.1 | 13.0 | 5.9 | 5.0 | 6.9 | 2.0 | 1.5 | 2.6 | 0.7 | 0.4 | 0.9 | yes | 3,713 |

CI - Confidence interval.

* Expected prevalence of double burden of malnutrition at individual level, calculated as the stunting prevalence multiplied by the overweight prevalence of each country.

† Significant prevalence of double burden at individual level was considered when the lower limit of confidence interval of the observed prevalence was higher than the upper limit of confidence interval of the prevalence expected by chance.

**Supplementary Table 2.** Prevalence of stunting, overweight and double burden at individual level, in children under five years from LMIC, by wealth quintiles.

| **Country** | **Year** | **Poorest** | | | | | **Second** | | | | | **Middle** | | | | | **Fourth** | | | | | **Wealthiest** | | | | |
| --- | --- | --- | --- | --- | --- | --- | --- | --- | --- | --- | --- | --- | --- | --- | --- | --- | --- | --- | --- | --- | --- | --- | --- | --- | --- | --- |
|  |  | **ST** | **OW** | **Both** | | | **ST** | **OW** | **Both** | | | **ST** | **OW** | **Both** | | | **ST** | **OW** | **Both** | | | **ST** | **OW** | **Both** | | |
|  |  | **%** | **%** | **%** | **95%CI** | | **%** | **%** | **%** | **95%CI** | | **%** | **%** | **%** | **95%CI** | | **%** | **%** | **%** | **95%CI** | | **%** | **%** | **%** | **95%CI** | |
| Albania | 2008 | 27.0 | 28.2 | 13.7 | 9.7 | 19.0 | 15.5 | 16.2 | 7.1 | 4.3 | 11.3 | 18.9 | 21.7 | 9.8 | 6.3 | 15.0 | 20.6 | 19.2 | 9.4 | 6.3 | 13.9 | 13.3 | 22.8 | 4.6 | 2.1 | 9.5 |
| Algeria | 2012 | 12.6 | 11.5 | 2.8 | 2.1 | 3.7 | 12.1 | 11.4 | 3.1 | 2.4 | 4.0 | 11.0 | 11.5 | 3.8 | 2.9 | 5.0 | 11.7 | 14.8 | 4.7 | 3.7 | 5.9 | 10.6 | 13.1 | 3.7 | 2.8 | 4.9 |
| Angola | 2015 | 47.3 | 2.5 | 1.3 | 0.8 | 2.2 | 45.1 | 2.5 | 1.1 | 0.7 | 1.7 | 38.8 | 3.6 | 1.6 | 0.9 | 2.8 | 26.6 | 4.0 | 1.3 | 0.6 | 3.1 | 20.4 | 4.5 | 1.5 | 0.6 | 3.7 |
| Armenia | 2015 | 12.0 | 16.7 | 4.3 | 2.5 | 7.2 | 11.5 | 16.0 | 2.5 | 1.2 | 5.1 | 11.5 | 15.0 | 4.5 | 2.4 | 8.1 | 6.2 | 12.5 | 3.1 | 1.5 | 6.5 | 5.9 | 8.5 | 2.1 | 1.0 | 4.2 |
| Azerbaijan | 2006 | 33.2 | 11.4 | 8.7 | 5.7 | 13.2 | 30.5 | 13.2 | 9.7 | 6.4 | 14.4 | 25.7 | 11.6 | 9.5 | 6.6 | 13.6 | 14.9 | 11.4 | 6.3 | 3.2 | 12.1 | 15.2 | 18.5 | 8.1 | 5.3 | 12.4 |
| Bangladesh | 2014 | 49.2 | 0.5 | 0.4 | 0.2 | 0.8 | 42.2 | 0.9 | 0.8 | 0.4 | 1.7 | 36.0 | 2.2 | 1.7 | 0.6 | 4.7 | 31.0 | 0.7 | 0.2 | 0.1 | 0.5 | 19.4 | 3.0 | 0.6 | 0.3 | 1.2 |
| Belarus | 2005 | 10.4 | 11.4 | 1.9 | 0.9 | 3.8 | 4.9 | 11.1 | 0.8 | 0.3 | 1.9 | 3.2 | 9.4 | 1.1 | 0.5 | 2.2 | 3.1 | 8.9 | 0.5 | 0.2 | 1.5 | 2.1 | 8.3 | 0.8 | 0.4 | 1.8 |
| Belize | 2015 | 26.1 | 3.8 | 1.2 | 0.6 | 2.4 | 14.5 | 6.8 | 0.9 | 0.3 | 2.7 | 12.4 | 8.4 | 0.3 | 0.1 | 1.3 | 10.4 | 11.0 | 0.2 | 0.0 | 1.3 | 5.4 | 7.8 | 0.2 | 0.0 | 1.3 |
| Benin | 2014 | 46.3 | 1.8 | 1.2 | 0.8 | 1.8 | 39.0 | 1.8 | 0.8 | 0.5 | 1.2 | 34.9 | 1.5 | 0.7 | 0.4 | 1.2 | 29.0 | 1.8 | 0.4 | 0.2 | 1.0 | 17.9 | 1.7 | 0.3 | 0.2 | 0.7 |
| Bhutan | 2010 | 41.4 | 6.2 | 3.1 | 2.1 | 4.4 | 39.9 | 7.1 | 4.0 | 2.8 | 5.7 | 38.4 | 8.4 | 6.5 | 4.9 | 8.5 | 27.6 | 6.3 | 3.7 | 2.6 | 5.1 | 21.4 | 10.2 | 6.0 | 4.2 | 8.3 |
| Bolivia | 2008 | 45.9 | 7.3 | 3.0 | 2.3 | 4.1 | 34.2 | 8.2 | 3.3 | 2.5 | 4.5 | 21.7 | 8.0 | 2.6 | 1.8 | 3.7 | 14.0 | 9.2 | 1.5 | 1.0 | 2.4 | 6.5 | 11.3 | 1.2 | 0.6 | 2.5 |
| Bosnia Herzegovina | 2011 | 10.1 | 13.6 | 4.9 | 2.2 | 10.3 | 9.6 | 15.4 | 2.9 | 1.7 | 5.0 | 6.8 | 15.7 | 3.1 | 1.7 | 5.6 | 7.6 | 20.7 | 4.9 | 2.0 | 11.3 | 10.3 | 20.7 | 4.1 | 2.3 | 7.1 |
| Burkina Faso | 2010 | 41.9 | 2.0 | 1.3 | 0.8 | 2.3 | 37.0 | 2.7 | 1.3 | 0.8 | 2.1 | 37.7 | 1.7 | 1.2 | 0.7 | 2.1 | 33.2 | 2.5 | 1.9 | 1.2 | 3.0 | 18.6 | 3.2 | 1.3 | 0.6 | 2.7 |
| Burundi | 2016 | 69.1 | 0.9 | 0.5 | 0.2 | 1.1 | 63.7 | 1.5 | 0.8 | 0.4 | 1.6 | 60.2 | 1.1 | 0.8 | 0.4 | 1.6 | 49.7 | 1.7 | 1.1 | 0.6 | 2.0 | 31.2 | 1.7 | 0.5 | 0.2 | 1.1 |
| CAR | 2010 | 45.3 | 1.6 | 1.1 | 0.7 | 1.9 | 44.7 | 2.2 | 1.3 | 0.8 | 2.1 | 41.4 | 1.3 | 0.6 | 0.3 | 1.1 | 39.4 | 1.9 | 0.9 | 0.5 | 1.7 | 30.3 | 2.2 | 0.8 | 0.4 | 1.8 |
| Cambodia | 2014 | 41.9 | 1.1 | 1.0 | 0.6 | 1.7 | 37.1 | 2.1 | 1.1 | 0.6 | 2.1 | 31.7 | 1.3 | 0.4 | 0.1 | 1.0 | 29.1 | 2.4 | 1.1 | 0.6 | 2.2 | 18.4 | 3.3 | 0.7 | 0.3 | 1.4 |
| Cameroon | 2014 | 41.6 | 2.6 | 1.1 | 0.6 | 2.0 | 41.2 | 5.0 | 2.8 | 1.9 | 4.2 | 31.3 | 7.9 | 2.4 | 1.6 | 3.5 | 21.8 | 9.2 | 2.2 | 1.5 | 3.1 | 14.5 | 11.2 | 2.4 | 1.4 | 3.8 |
| Chad | 2014 | 41.2 | 2.5 | 1.3 | 0.8 | 2.0 | 39.8 | 3.0 | 1.6 | 1.0 | 2.3 | 40.4 | 2.8 | 1.4 | 0.9 | 2.1 | 44.7 | 2.0 | 0.9 | 0.5 | 1.4 | 31.5 | 2.4 | 1.2 | 0.7 | 1.8 |
| Colombia | 2010 | 19.4 | 3.5 | 0.6 | 0.4 | 0.9 | 13.2 | 4.7 | 0.4 | 0.2 | 0.7 | 11.8 | 4.5 | 0.4 | 0.2 | 0.8 | 9.8 | 5.4 | 0.7 | 0.4 | 1.4 | 6.8 | 7.3 | 0.5 | 0.2 | 1.4 |
| Comoros | 2012 | 38.2 | 7.1 | 4.6 | 3.2 | 6.7 | 32.5 | 8.8 | 4.4 | 2.6 | 7.4 | 25.9 | 9.0 | 4.8 | 3.0 | 7.7 | 27.0 | 13.2 | 6.4 | 3.8 | 10.7 | 21.9 | 9.8 | 4.2 | 2.2 | 7.8 |
| Congo (Brazzaville) | 2014 | 30.7 | 2.2 | 1.2 | 0.8 | 1.6 | 24.4 | 6.9 | 2.4 | 1.2 | 4.6 | 18.2 | 6.1 | 2.4 | 1.5 | 3.8 | 13.9 | 6.3 | 1.2 | 0.6 | 2.5 | 14.6 | 9.7 | 3.4 | 1.7 | 6.5 |
| Congo DR | 2013 | 49.7 | 4.6 | 2.9 | 2.1 | 4.1 | 48.3 | 3.1 | 2.1 | 1.4 | 3.2 | 45.8 | 3.9 | 2.7 | 1.7 | 4.2 | 41.4 | 4.7 | 2.7 | 1.9 | 4.0 | 22.9 | 4.4 | 1.7 | 0.9 | 3.0 |
| Cote d’Ivoire | 2016 | 30.3 | 1.4 | 0.6 | 0.3 | 1.1 | 28.5 | 1.5 | 0.8 | 0.4 | 1.5 | 22.0 | 1.8 | 0.9 | 0.5 | 1.7 | 11.1 | 0.5 | 0.0 | 0.0 | 0.2 | 8.7 | 2.3 | 0.5 | 0.1 | 2.3 |
| Dominican Republic | 2013 | 11.3 | 3.5 | 0.9 | 0.4 | 1.9 | 8.8 | 6.6 | 1.6 | 0.8 | 3.2 | 3.9 | 8.1 | 1.4 | 0.7 | 3.0 | 4.9 | 9.0 | 0.8 | 0.3 | 2.3 | 3.9 | 10.9 | 0.0 | 0.0 | 0.0 |
| Egypt | 2014 | 24.1 | 15.6 | 8.4 | 6.9 | 10.2 | 23.1 | 12.8 | 7.0 | 5.7 | 8.5 | 18.1 | 13.9 | 6.7 | 5.5 | 8.1 | 20.0 | 15.0 | 7.4 | 6.1 | 9.0 | 23.4 | 17.7 | 8.6 | 6.9 | 10.7 |
| El Salvador | 2014 | 23.6 | 3.9 | 1.3 | 0.8 | 2.1 | 13.2 | 4.6 | 0.2 | 0.1 | 0.6 | 12.1 | 8.4 | 0.4 | 0.1 | 1.2 | 9.4 | 9.4 | 0.1 | 0.0 | 0.4 | 5.3 | 6.6 | 0.2 | 0.1 | 0.6 |
| Eswatini | 2014 | 30.2 | 5.8 | 1.8 | 1.0 | 3.4 | 31.2 | 7.0 | 2.2 | 1.3 | 3.7 | 27.8 | 7.6 | 2.4 | 1.3 | 4.5 | 23.3 | 9.9 | 1.4 | 0.5 | 3.8 | 9.2 | 17.5 | 1.8 | 0.8 | 4.0 |
| Ethiopia | 2016 | 44.6 | 3.4 | 1.7 | 0.8 | 3.6 | 42.8 | 2.2 | 1.2 | 0.6 | 2.3 | 37.9 | 2.1 | 1.1 | 0.6 | 2.1 | 35.4 | 3.5 | 1.8 | 1.1 | 3.0 | 25.6 | 3.1 | 0.6 | 0.3 | 1.4 |
| Gabon | 2012 | 30.0 | 5.9 | 2.3 | 1.7 | 3.2 | 18.8 | 8.3 | 2.7 | 1.6 | 4.7 | 12.3 | 6.7 | 2.1 | 1.0 | 4.3 | 11.9 | 8.9 | 2.7 | 1.3 | 5.7 | 5.8 | 6.9 | 1.6 | 0.6 | 4.3 |
| Gambia | 2013 | 29.4 | 2.2 | 1.4 | 0.7 | 3.0 | 27.2 | 3.3 | 1.9 | 1.0 | 3.5 | 25.2 | 2.5 | 1.7 | 0.9 | 3.1 | 22.4 | 3.4 | 3.0 | 1.6 | 5.5 | 15.2 | 2.0 | 0.6 | 0.1 | 2.5 |
| Georgia | 2005 | 22.0 | 24.9 | 9.5 | 6.4 | 14.0 | 16.5 | 20.2 | 6.5 | 3.9 | 10.8 | 15.5 | 23.2 | 8.3 | 5.4 | 12.6 | 13.3 | 20.1 | 5.7 | 3.4 | 9.5 | 8.2 | 17.1 | 3.7 | 1.9 | 6.9 |
| Ghana | 2014 | 24.8 | 1.5 | 0.8 | 0.3 | 1.6 | 25.5 | 3.3 | 1.9 | 0.7 | 5.3 | 17.9 | 2.0 | 0.8 | 0.3 | 2.0 | 14.4 | 2.1 | 0.2 | 0.0 | 1.5 | 8.5 | 4.6 | 0.6 | 0.2 | 2.1 |
| Guatemala | 2014 | 65.9 | 4.3 | 2.1 | 1.6 | 2.8 | 57.1 | 3.9 | 1.9 | 1.4 | 2.6 | 43.8 | 4.4 | 1.1 | 0.8 | 1.7 | 28.9 | 4.7 | 0.7 | 0.4 | 1.2 | 17.4 | 7.4 | 1.0 | 0.5 | 1.8 |
| Guinea | 2016 | 39.1 | 3.2 | 1.6 | 1.0 | 2.3 | 39.6 | 5.4 | 2.8 | 2.0 | 4.0 | 34.5 | 4.0 | 1.6 | 1.0 | 2.6 | 26.8 | 3.7 | 1.5 | 0.9 | 2.4 | 18.4 | 3.4 | 0.9 | 0.5 | 1.7 |
| Guinea Bissau | 2014 | 30.7 | 2.4 | 0.8 | 0.5 | 1.2 | 31.4 | 2.0 | 1.1 | 0.7 | 1.9 | 32.8 | 1.8 | 0.6 | 0.3 | 1.2 | 22.7 | 1.6 | 0.6 | 0.3 | 1.3 | 14.6 | 4.6 | 1.1 | 0.4 | 3.4 |
| Guyana | 2014 | 21.0 | 5.1 | 1.7 | 1.0 | 2.9 | 11.6 | 4.0 | 1.7 | 0.7 | 4.0 | 7.3 | 5.0 | 0.2 | 0.0 | 1.5 | 5.6 | 5.5 | 0.2 | 0.0 | 1.7 | 7.2 | 7.6 | 0.9 | 0.2 | 2.9 |
| Haiti | 2016 | 33.9 | 2.7 | 1.4 | 0.8 | 2.3 | 23.9 | 3.1 | 1.0 | 0.6 | 1.6 | 17.4 | 3.2 | 0.6 | 0.3 | 1.2 | 16.7 | 2.9 | 0.2 | 0.1 | 1.1 | 9.1 | 5.0 | 0.7 | 0.3 | 1.8 |
| Honduras | 2011 | 42.1 | 3.5 | 0.9 | 0.6 | 1.3 | 25.1 | 3.9 | 0.7 | 0.4 | 1.1 | 16.4 | 4.4 | 0.4 | 0.2 | 0.9 | 11.5 | 6.6 | 0.6 | 0.3 | 1.1 | 8.0 | 9.1 | 0.1 | 0.0 | 1.0 |
| India | 2015 | 50.9 | 1.8 | 1.2 | 1.1 | 1.3 | 43.0 | 1.9 | 1.1 | 1.0 | 1.2 | 36.0 | 2.3 | 1.3 | 1.2 | 1.4 | 28.9 | 2.8 | 1.6 | 1.4 | 1.8 | 22.0 | 3.7 | 1.7 | 1.5 | 2.0 |
| Iraq | 2011 | 24.4 | 8.6 | 4.5 | 3.9 | 5.1 | 22.8 | 11.2 | 5.3 | 4.6 | 6.2 | 20.0 | 11.2 | 4.8 | 4.0 | 5.7 | 21.1 | 12.7 | 6.2 | 5.1 | 7.5 | 21.2 | 14.7 | 6.8 | 5.5 | 8.5 |
| Jordan | 2012 | 13.8 | 4.8 | 0.9 | 0.5 | 1.9 | 7.0 | 3.3 | 0.4 | 0.2 | 0.8 | 6.2 | 3.3 | 0.7 | 0.3 | 1.4 | 6.8 | 4.7 | 0.3 | 0.1 | 0.6 | 1.8 | 6.9 | 0.1 | 0.0 | 0.6 |
| Kazakhstan | 2015 | 10.3 | 7.7 | 2.1 | 1.2 | 3.6 | 8.1 | 7.0 | 1.6 | 0.9 | 2.9 | 8.3 | 8.9 | 2.1 | 1.2 | 3.5 | 6.9 | 11.6 | 2.5 | 1.5 | 4.1 | 6.2 | 12.5 | 1.5 | 0.7 | 3.2 |
| Kenya | 2008 | 44.4 | 4.2 | 3.0 | 2.0 | 4.4 | 39.2 | 4.3 | 2.4 | 1.4 | 3.9 | 34.4 | 4.0 | 2.1 | 1.2 | 3.5 | 29.1 | 5.7 | 2.7 | 1.7 | 4.4 | 24.5 | 5.5 | 1.8 | 1.0 | 3.2 |
| Kosovo | 2013 | 8.9 | 3.6 | 0.2 | 0.0 | 1.6 | 4.3 | 2.8 | 0.3 | 0.0 | 2.3 | 2.6 | 3.8 | 0.4 | 0.0 | 2.5 | 3.0 | 4.0 | 0.0 | 0.0 | 0.0 | 0.7 | 7.9 | 0.0 | 0.0 | 0.0 |
| Kyrgyzstan | 2014 | 17.7 | 6.2 | 2.5 | 1.6 | 3.9 | 14.2 | 4.4 | 0.4 | 0.2 | 1.2 | 10.1 | 7.0 | 1.4 | 0.7 | 2.5 | 10.7 | 9.2 | 2.1 | 1.0 | 4.2 | 10.7 | 9.2 | 2.5 | 1.4 | 4.3 |
| Lao | 2011 | 60.6 | 2.0 | 1.3 | 0.9 | 1.9 | 50.2 | 2.5 | 1.3 | 0.9 | 1.9 | 41.9 | 1.7 | 0.9 | 0.5 | 1.5 | 31.7 | 2.0 | 0.9 | 0.5 | 1.6 | 19.7 | 2.1 | 0.6 | 0.3 | 1.1 |
| Lesotho | 2014 | 45.6 | 7.5 | 3.8 | 2.3 | 6.2 | 38.1 | 7.3 | 2.9 | 1.7 | 5.1 | 34.8 | 7.4 | 3.3 | 1.6 | 6.9 | 28.2 | 7.3 | 1.4 | 0.6 | 3.5 | 13.4 | 7.4 | 1.1 | 0.3 | 3.3 |
| Liberia | 2013 | 35.3 | 3.1 | 1.7 | 1.1 | 2.9 | 35.2 | 2.2 | 1.4 | 0.7 | 2.5 | 35.3 | 3.1 | 1.1 | 0.3 | 3.4 | 27.7 | 2.0 | 1.1 | 0.4 | 3.3 | 19.9 | 4.3 | 1.9 | 0.7 | 5.2 |
| Malawi | 2015 | 45.7 | 4.1 | 2.2 | 1.4 | 3.3 | 40.4 | 4.8 | 3.1 | 1.9 | 4.9 | 36.8 | 4.8 | 1.7 | 1.0 | 3.0 | 33.1 | 4.3 | 1.4 | 0.8 | 2.5 | 24.3 | 4.9 | 1.4 | 0.7 | 2.6 |
| Maldives | 2009 | 21.9 | 4.3 | 0.9 | 0.4 | 2.1 | 23.1 | 6.9 | 2.1 | 1.2 | 3.8 | 17.6 | 4.9 | 1.1 | 0.5 | 2.4 | 15.4 | 7.0 | 1.5 | 0.6 | 3.5 | 15.7 | 7.0 | 1.8 | 0.7 | 4.8 |
| Mali | 2015 | 40.5 | 1.9 | 1.4 | 1.0 | 2.0 | 36.2 | 1.8 | 1.1 | 0.6 | 2.1 | 33.5 | 1.5 | 0.9 | 0.5 | 1.5 | 25.1 | 2.0 | 0.9 | 0.5 | 1.5 | 15.2 | 2.5 | 0.9 | 0.5 | 1.6 |
| Mauritania | 2015 | 36.6 | 0.7 | 0.4 | 0.2 | 0.9 | 31.1 | 0.9 | 0.3 | 0.1 | 0.8 | 27.4 | 0.8 | 0.3 | 0.1 | 0.6 | 23.7 | 2.0 | 0.6 | 0.2 | 1.3 | 18.7 | 2.4 | 1.1 | 0.6 | 1.9 |
| Mexico | 2015 | 22.9 | 4.1 | 0.4 | 0.2 | 0.8 | 12.2 | 6.2 | 0.8 | 0.4 | 2.0 | 9.8 | 6.0 | 0.1 | 0.0 | 0.3 | 6.4 | 5.0 | 0.1 | 0.0 | 0.4 | 4.9 | 4.1 | 0.1 | 0.0 | 0.7 |
| Moldova | 2012 | 11.4 | 2.9 | 0.4 | 0.1 | 2.7 | 5.6 | 2.7 | 0.3 | 0.0 | 2.3 | 9.1 | 7.5 | 1.5 | 0.5 | 4.4 | 3.4 | 4.4 | 0.2 | 0.0 | 1.7 | 2.7 | 6.6 | 0.6 | 0.2 | 1.7 |
| Mongolia | 2013 | 18.6 | 9.6 | 2.0 | 1.4 | 3.0 | 12.5 | 10.4 | 2.0 | 1.3 | 3.0 | 8.5 | 9.1 | 0.9 | 0.5 | 1.7 | 7.7 | 11.7 | 1.3 | 0.7 | 2.2 | 5.7 | 11.7 | 1.3 | 0.8 | 2.3 |
| Montenegro | 2013 | 4.7 | 15.8 | 2.7 | 1.1 | 6.3 | 9.7 | 26.3 | 8.9 | 3.2 | 22.3 | 10.6 | 20.3 | 5.3 | 2.7 | 10.2 | 12.8 | 22.8 | 8.6 | 4.6 | 15.4 | 8.6 | 25.5 | 2.9 | 1.4 | 6.0 |
| Mozambique | 2011 | 51.1 | 5.6 | 4.3 | 3.3 | 5.6 | 48.0 | 7.3 | 5.2 | 3.9 | 7.0 | 46.4 | 7.9 | 5.3 | 3.9 | 7.0 | 37.4 | 8.5 | 4.7 | 3.5 | 6.2 | 24.1 | 8.0 | 2.9 | 2.2 | 3.8 |
| Myanmar | 2015 | 38.0 | 0.7 | 0.1 | 0.0 | 0.4 | 31.9 | 1.2 | 1.0 | 0.4 | 2.5 | 29.1 | 1.3 | 0.7 | 0.2 | 1.9 | 21.1 | 2.0 | 0.3 | 0.1 | 1.3 | 16.0 | 1.9 | 0.5 | 0.1 | 2.4 |
| Namibia | 2013 | 31.3 | 2.2 | 0.7 | 0.2 | 2.0 | 28.7 | 2.0 | 1.4 | 0.7 | 2.8 | 24.1 | 3.8 | 1.2 | 0.5 | 2.7 | 16.7 | 5.5 | 0.8 | 0.2 | 2.8 | 8.7 | 4.3 | 0.2 | 0.0 | 1.3 |
| Nepal | 2016 | 49.2 | 1.4 | 0.4 | 0.1 | 1.1 | 38.7 | 1.1 | 0.3 | 0.1 | 1.3 | 35.7 | 0.2 | 0.0 | 0.0 | 0.2 | 32.4 | 0.7 | 0.2 | 0.0 | 1.3 | 16.5 | 3.7 | 1.7 | 0.7 | 4.2 |
| Niger | 2012 | 46.9 | 2.3 | 1.6 | 1.0 | 2.7 | 48.0 | 2.4 | 1.4 | 0.7 | 2.7 | 41.8 | 1.7 | 0.8 | 0.2 | 2.3 | 46.7 | 2.5 | 1.2 | 0.7 | 2.1 | 34.5 | 3.0 | 1.7 | 0.9 | 3.2 |
| Nigeria | 2016 | 62.8 | 1.5 | 1.1 | 0.8 | 1.4 | 54.5 | 1.9 | 1.0 | 0.7 | 1.5 | 43.5 | 1.4 | 1.0 | 0.7 | 1.4 | 32.2 | 1.3 | 0.6 | 0.4 | 0.8 | 18.3 | 1.4 | 0.5 | 0.3 | 0.9 |
| North Macedonia | 2011 | 7.2 | 4.3 | 0.5 | 0.1 | 2.3 | 5.8 | 9.6 | 1.2 | 0.4 | 3.2 | 4.7 | 22.2 | 2.1 | 0.9 | 4.9 | 4.6 | 14.2 | 0.6 | 0.2 | 2.0 | 2.0 | 14.2 | 0.7 | 0.2 | 2.4 |
| Pakistan | 2012 | 61.6 | 2.8 | 2.0 | 1.3 | 3.3 | 55.7 | 3.2 | 2.8 | 1.6 | 5.0 | 40.7 | 2.6 | 1.7 | 0.8 | 3.5 | 37.8 | 2.9 | 1.8 | 1.0 | 3.3 | 23.0 | 5.0 | 3.2 | 2.0 | 5.2 |
| Paraguay | 2016 | 12.8 | 8.8 | 0.7 | 0.2 | 2.3 | 5.6 | 12.4 | 0.7 | 0.3 | 1.6 | 4.0 | 11.1 | 0.1 | 0.0 | 0.5 | 3.2 | 15.8 | 1.1 | 0.5 | 2.1 | 0.8 | 16.8 | 0.2 | 0.1 | 0.9 |
| Peru | 2012 | 38.5 | 4.2 | 1.1 | 0.7 | 1.8 | 20.4 | 3.5 | 0.3 | 0.1 | 0.5 | 11.3 | 6.2 | 0.3 | 0.1 | 0.8 | 5.3 | 11.0 | 0.2 | 0.1 | 1.0 | 3.4 | 15.9 | 0.1 | 0.0 | 0.5 |
| Rwanda | 2014 | 48.6 | 6.9 | 3.2 | 2.2 | 4.5 | 44.7 | 7.2 | 2.8 | 1.8 | 4.3 | 37.5 | 6.1 | 3.0 | 1.9 | 4.6 | 30.2 | 8.7 | 3.3 | 2.1 | 5.1 | 20.9 | 10.3 | 2.4 | 1.4 | 4.0 |
| Sao Tome and Principe | 2014 | 25.5 | 1.9 | 0.9 | 0.4 | 2.1 | 20.2 | 1.8 | 0.4 | 0.1 | 1.5 | 18.3 | 2.5 | 1.5 | 0.6 | 3.5 | 12.0 | 3.4 | 2.1 | 0.7 | 5.8 | 6.8 | 2.4 | 0.0 | 0.0 | 0.0 |
| Senegal | 2016 | 25.6 | 0.4 | 0.0 | 0.0 | 0.3 | 19.1 | 1.0 | 0.1 | 0.0 | 0.6 | 17.0 | 1.1 | 0.3 | 0.1 | 0.8 | 11.7 | 0.6 | 0.2 | 0.0 | 1.1 | 6.3 | 2.0 | 0.1 | 0.0 | 0.4 |
| Senegal | 2017 | 27.6 | 1.2 | 0.4 | 0.2 | 0.8 | 19.0 | 0.6 | 0.2 | 0.1 | 0.5 | 14.5 | 0.5 | 0.2 | 0.1 | 0.6 | 9.6 | 1.0 | 0.3 | 0.1 | 0.9 | 6.2 | 1.5 | 0.1 | 0.0 | 0.4 |
| Serbia | 2014 | 13.6 | 16.4 | 2.1 | 0.8 | 5.4 | 3.6 | 17.2 | 1.2 | 0.5 | 2.5 | 7.2 | 12.8 | 3.6 | 1.5 | 8.2 | 3.3 | 11.7 | 1.6 | 0.6 | 3.9 | 4.1 | 12.8 | 2.4 | 1.3 | 4.5 |
| Sierra Leone | 2013 | 42.6 | 8.7 | 4.6 | 3.5 | 6.0 | 40.4 | 6.0 | 4.0 | 2.7 | 5.8 | 38.1 | 7.7 | 3.9 | 2.6 | 5.6 | 35.0 | 7.1 | 3.2 | 2.2 | 4.4 | 28.1 | 8.4 | 6.2 | 4.2 | 9.1 |
| Somalia | 2006 | 51.9 | 5.7 | 2.5 | 1.8 | 3.5 | 51.8 | 4.6 | 1.8 | 1.2 | 2.8 | 47.2 | 4.7 | 2.9 | 1.8 | 4.6 | 32.8 | 5.2 | 2.4 | 1.4 | 4.1 | 25.7 | 3.2 | 1.5 | 0.9 | 2.5 |
| South Sudan | 2010 | 31.3 | 4.8 | 3.3 | 2.3 | 4.8 | 34.1 | 6.2 | 3.1 | 2.1 | 4.6 | 32.0 | 6.7 | 4.0 | 2.7 | 5.9 | 31.7 | 7.2 | 4.1 | 2.8 | 6.0 | 26.5 | 4.9 | 2.6 | 1.7 | 4.0 |
| St Lucia | 2012 | 5.2 | 5.1 | 2.2 | 0.3 | 14.8 | 1.7 | 8.4 | 0.0 | 0.0 | 0.0 | 4.1 | 4.6 | 0.0 | 0.0 | 0.0 | 0.9 | 4.5 | 0.0 | 0.0 | 0.0 | 0.0 | 8.9 | 0.0 | 0.0 | 0.0 |
| State of Palestine | 2014 | 7.3 | 6.3 | 0.5 | 0.2 | 0.9 | 8.4 | 6.7 | 0.5 | 0.2 | 1.2 | 7.6 | 10.0 | 1.1 | 0.7 | 1.8 | 7.3 | 9.4 | 2.4 | 1.6 | 3.6 | 5.8 | 9.6 | 2.2 | 1.4 | 3.4 |
| Sudan | 2014 | 44.0 | 1.4 | 0.8 | 0.4 | 1.3 | 47.3 | 2.0 | 1.2 | 0.8 | 1.8 | 43.6 | 3.4 | 2.2 | 1.4 | 3.4 | 33.8 | 3.8 | 2.5 | 1.2 | 5.0 | 21.1 | 4.8 | 2.5 | 1.3 | 4.9 |
| Suriname | 2010 | 13.4 | 3.1 | 0.8 | 0.4 | 1.6 | 8.3 | 2.4 | 0.5 | 0.1 | 2.2 | 5.1 | 6.0 | 0.0 | 0.0 | 0.0 | 6.6 | 4.6 | 0.0 | 0.0 | 0.0 | 5.5 | 5.6 | 1.9 | 0.7 | 5.3 |
| Syria | 2006 | 36.1 | 20.4 | 13.6 | 11.5 | 16.1 | 26.9 | 17.4 | 9.3 | 8.0 | 10.7 | 28.0 | 17.5 | 10.1 | 8.7 | 11.6 | 26.2 | 19.0 | 10.1 | 8.4 | 12.1 | 26.4 | 20.0 | 11.7 | 9.9 | 13.8 |
| Tajikistan | 2012 | 32.1 | 4.3 | 1.8 | 0.9 | 3.4 | 29.0 | 3.3 | 2.0 | 1.2 | 3.2 | 23.4 | 7.3 | 3.5 | 2.0 | 6.1 | 24.9 | 8.1 | 4.7 | 3.1 | 7.1 | 20.9 | 6.4 | 4.0 | 2.7 | 5.8 |
| Tanzania | 2015 | 39.9 | 2.6 | 1.4 | 0.9 | 1.9 | 39.4 | 3.4 | 1.6 | 1.0 | 2.4 | 38.7 | 4.3 | 1.9 | 1.3 | 2.8 | 29.7 | 3.7 | 1.6 | 1.1 | 2.5 | 19.2 | 4.7 | 1.1 | 0.7 | 1.8 |
| Thailand | 2015 | 13.0 | 7.5 | 1.3 | 0.4 | 3.7 | 10.9 | 7.5 | 1.2 | 0.6 | 2.4 | 9.4 | 8.2 | 1.4 | 0.6 | 3.2 | 8.0 | 9.8 | 0.7 | 0.4 | 1.3 | 11.8 | 8.0 | 1.8 | 0.9 | 3.4 |
| Timor-Leste | 2016 | 48.0 | 5.0 | 3.2 | 2.3 | 4.4 | 49.1 | 5.4 | 3.5 | 2.5 | 4.8 | 48.6 | 6.2 | 3.9 | 2.7 | 5.5 | 45.1 | 4.1 | 2.6 | 1.8 | 3.9 | 36.3 | 6.6 | 4.6 | 3.0 | 7.0 |
| Togo | 2013 | 33.4 | 2.1 | 0.9 | 0.5 | 1.7 | 37.5 | 2.0 | 1.1 | 0.5 | 2.7 | 32.5 | 2.0 | 1.0 | 0.4 | 2.4 | 19.4 | 1.1 | 0.6 | 0.2 | 2.4 | 10.6 | 2.5 | 0.3 | 0.1 | 1.2 |
| Tunisia | 2011 | 15.6 | 14.8 | 5.3 | 3.4 | 8.1 | 10.2 | 12.5 | 2.5 | 1.3 | 4.6 | 7.5 | 15.7 | 2.7 | 1.4 | 5.0 | 9.1 | 16.0 | 3.8 | 2.3 | 6.2 | 7.7 | 12.3 | 0.9 | 0.4 | 2.3 |
| Turkmenistan | 2015 | 15.5 | 4.8 | 2.6 | 1.6 | 4.3 | 9.7 | 6.1 | 2.3 | 1.3 | 4.0 | 9.7 | 7.3 | 1.4 | 0.7 | 2.5 | 10.8 | 5.7 | 1.9 | 1.1 | 3.2 | 11.4 | 5.4 | 1.5 | 0.8 | 2.8 |
| Uganda | 2016 | 32.3 | 2.7 | 1.1 | 0.6 | 2.1 | 33.2 | 4.0 | 1.1 | 0.6 | 2.0 | 33.0 | 4.6 | 1.5 | 0.9 | 2.4 | 27.2 | 3.9 | 1.1 | 0.6 | 2.1 | 16.7 | 3.4 | 0.8 | 0.3 | 2.3 |
| Uzbekistan | 2006 | 21.3 | 11.3 | 3.8 | 2.7 | 5.3 | 22.3 | 12.3 | 4.2 | 3.0 | 5.9 | 20.3 | 13.3 | 4.9 | 3.6 | 6.7 | 17.8 | 12.4 | 4.2 | 3.0 | 5.9 | 15.6 | 11.9 | 4.0 | 2.9 | 5.5 |
| Vanuatu | 2007 | 28.6 | 4.2 | 2.9 | 1.2 | 6.5 | 26.0 | 2.9 | 1.8 | 0.8 | 4.3 | 26.1 | 5.2 | 1.7 | 0.7 | 4.4 | 23.7 | 5.4 | 2.6 | 1.1 | 5.9 | 24.2 | 7.2 | 3.4 | 1.6 | 7.0 |
| Vietnam | 2010 | 40.9 | 1.6 | 0.9 | 0.4 | 2.1 | 24.2 | 2.8 | 0.4 | 0.2 | 1.2 | 24.2 | 2.9 | 0.5 | 0.2 | 1.3 | 15.6 | 6.3 | 0.9 | 0.4 | 2.0 | 6.1 | 8.9 | 0.7 | 0.2 | 2.0 |
| Yemen | 2013 | 59.2 | 1.3 | 1.2 | 0.7 | 1.9 | 55.5 | 2.0 | 1.6 | 1.2 | 2.3 | 48.1 | 1.9 | 1.3 | 0.9 | 2.0 | 38.3 | 2.8 | 1.4 | 1.0 | 2.0 | 25.9 | 2.4 | 1.1 | 0.7 | 1.7 |
| Zambia | 2013 | 47.3 | 5.3 | 3.2 | 2.5 | 4.0 | 41.7 | 4.9 | 2.5 | 1.8 | 3.3 | 40.2 | 6.2 | 3.7 | 2.9 | 4.6 | 37.7 | 6.1 | 3.8 | 2.8 | 5.2 | 28.4 | 6.7 | 3.4 | 2.2 | 5.2 |

ST: Stunting prevalence.

OW: Overweight prevalence.

**Supplementary Figure 1.** Scatter diagram of overweight prevalence according to stunting prevalence, for country income groups.


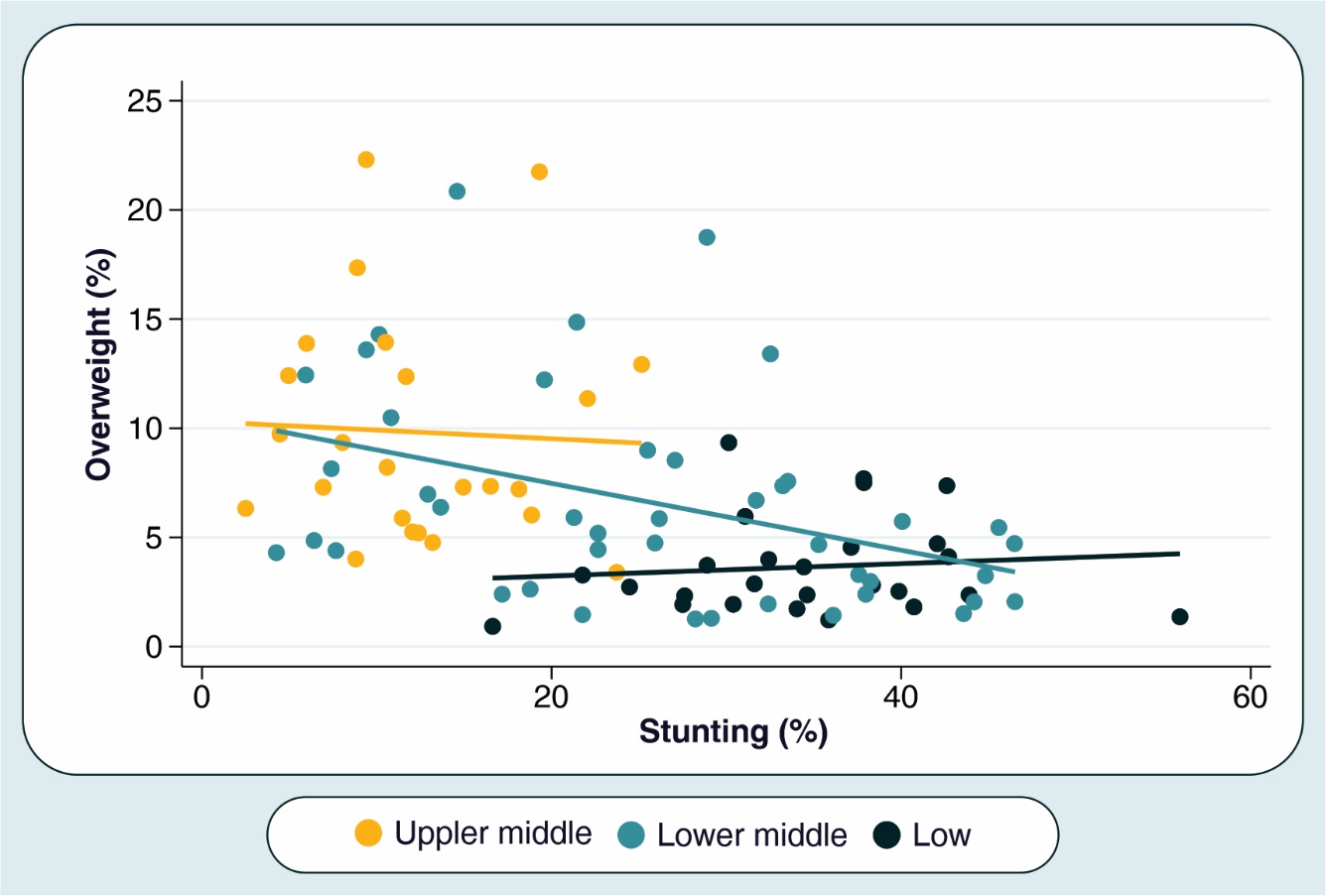

Supplement: Supplementary file 1 [file S1368980020001226sup001.docx]
